# Projecting Extinction Risk and Assessing Conservation Effectiveness for Three Threatened Relict Ferns in the Western Mediterranean Basin

**DOI:** 10.3390/plants14152380

**Published:** 2025-08-01

**Authors:** Ángel Enrique Salvo-Tierra, Jaime Francisco Pereña-Ortiz, Ángel Ruiz-Valero

**Affiliations:** Departament of Botany and Plant Physiology, Faculty of Sciences, University of Málaga, 29010 Málaga, Spain; salvo@uma.es (Á.E.S.-T.); arvalero@uma.es (Á.R.-V.)

**Keywords:** Monilophyta, protected areas, endangered species, survival rate, conservation, relict ferns

## Abstract

Relict fern species, confined to microhabitats with stable historical conditions, are especially vulnerable to climate change. The Alboran Arc hosts a unique relict fern flora, including *Culcita macrocarpa*, *Diplazium caudatum*, and *Pteris incompleta*, and functions as a major Pleistocene refuge. This study assesses the population trends and climate sensitivity of these species in Los Alcornocales Natural Park using annual abundance time series for a decade, empirical survival projections, and principal component analysis to identify key climatic drivers. Results reveal distinct climate response clusters among populations, though intra-specific variation highlights the importance of local conditions. Climate change is already impacting population viability, especially for *P. incompleta*, which shows high sensitivity to rising maximum temperatures and prolonged heatwaves. Climate-driven models forecast more severe declines than empirical ones, particularly for *C. macrocarpa* and *P. incompleta*, with the latter showing a projected collapse by the mid-century. In contrast, *D. caudatum* exhibits moderate vulnerability. Crucially, the divergence between models underscores the impact of conservation efforts: without reinforcement and reintroduction actions, projected declines would likely be more severe. These results project a decline in the populations of the studied ferns, highlighting the urgent need to continue implementing both in situ and ex situ conservation measures.

## 1. Introduction

Recently, the increasing extinction of species populations has been linked to the effects of climate change caused by ‘environmental noise’ generated by human activities [1]. However, it is a natural occurrence that population extinctions can occur at the interface of biotic and abiotic factors embedded in more dynamic processes at taxonomic range boundaries. As Purvis et al. [2] note, extinction is the final evolutionary process in the life of any species. In this sense, the extinction of populations must be seen as a natural occurrence in the history of biodiversity. This statement leads to a question of considerable interest: is the preservation of populations whose existence depends on changing environmental factors and competition with other organisms obligatory?

The extinction processes of populations have been analyzed by various authors, generally resulting in debates on the dynamics of these processes and on how to establish rules and guidelines that could be reflected in the elaboration of conservation programs [3,4,5,6,7]. Extinction risk is shaped by complex interactions among demographic, genetic, and environmental factors. While low population densities have long been associated with higher extinction risk, studies show that even high-density populations can collapse due to overcompensatory dynamics and elevated growth rates [8,9]. Genetic processes further amplify extinction risk through feedback loops like the extinction vortex, particularly in fragmented habitats [10]. Additionally, habitat loss and climate change—driven by human activities—exacerbate local population declines, often preceding species-wide extinctions [11]. Stochastic models reveal how external disturbances and catastrophic events further influence extinction outcomes, though their complexity may limit practical application in conservation [12,13].

Given the inherent complexity of evaluating the combined effects of demographic, genetic, and environmental factors, researchers have increasingly turned to more simplified approaches that bypass the need to assess each variable in detail. In this context, lists of threatened species have emerged as effective tools for projecting future extinctions, with rising threat levels serving as proxies for the estimated time to extinction [2]. As the biodiversity crisis intensifies, accurately predicting which species, and particularly which populations, are most at risk of extinction becomes an urgent challenge. To address this, several potential predictors of extinction over time have been proposed [14]. Moreover, the pressing need to anticipate the impacts of current climate change has highlighted the relevance of studies analyzing species’ responses to past climatic shifts [15].

Relict species, defined as taxonomic units that persist outside their current general distribution range, offer valuable insights into historical biogeographical patterns and species’ resilience to environmental change. Their continued existence implies a previously broader distribution and serves as a living archive of past climatic and ecological conditions [16], positioning them as key indicators for understanding long-term survival mechanisms and informing conservation strategies. These taxa are often remnants of populations that thrived under bioclimatic regimes markedly different from those of today, now confined to microhabitats with topoclimates resembling their original environmental niches. The persistence of such populations may reflect niche stability over geological timescales, while the extinction of other components of their former communities could be linked to greater sensitivity environmental changes. In this regard, contemporary demographic studies of relict species can provide critical data for interpreting and testing hypotheses derived from biogeographical analyses [15].

Due to their highly specific niche requirements, relict taxa are often confined to isolated microhabitats that preserve historical environmental conditions, making them particularly vulnerable to extinction driven by climate change and other anthropogenic pressures [17]. Their restriction to such narrow habitats increases their susceptibility to ongoing climatic shifts, as these environments, unlike their former broader ranges, offer limited opportunities for adaptation or spatial redistribution [18]. Local adaptation is essential for their persistence; however, the contraction of their ecological range and progressive habitat fragmentation exacerbates extinction risk by reducing genetic variability and undermining population resilience. Furthermore, Grandcolas et al. [19] observed that the extinction of most close relatives of relict taxa, combined with their geographic confinement to environmental refugia, constitutes a recurrent evolutionary pattern. This underscores the urgent need to study relict species not only as remnants of the past but also as critical models for understanding species survival under extreme ecological constraints.

A clear model of relict floras is that which characterizes the Macaronesian region, a biogeographical unit [20] that includes the archipelagos of the Azores, Madeira and the Canary Islands, and in some cases even the floras of Cape Verde and the continental enclaves of North Africa and the Iberian Peninsula, where species with Macaronesian affinities are found [21]. Current evidence both complements and, in some cases, challenges the traditional view of Macaronesia as a museum of Tertiary relicts, as framed by the relictism model and the Refugial Microhabitat Hypothesis. These frameworks posit that many relict taxa have persisted in the region due to the presence of microhabitats—such as laurel and cloud forests—with stable topoclimatic conditions that closely resemble their ancestral environments [22]. This theory interprets the Macaronesian flora, particularly its pteridophytes and laurel forest elements, as remnants of the Tertiary subtropical vegetation that once extended across southern Europe and North Africa [23,24,25,26]. However, recent studies indicate that the Macaronesian laurel forest flora represents a complex assemblage of both ancient and more recent lineages, with “Tertiary relict” status applying only to a subset of taxa [20,27,28,29]. Recent hypotheses emphasize the recent and dynamic assembly of the Macaronesian flora, shaped by high turnover rates, colonization, extinction, speciation, and long-distance dispersal, processes that were particularly intensified during the climatic fluctuations of the Pliocene and Pleistocene [27,30].

These findings collectively underscore that the Macaronesian flora cannot be explained by a single ancestral origin or static legacy, but rather by a combination of recent colonization, species turnover, and the persistence of relict lineages in stable microhabitats. This integrative perspective supports the idea of Macaronesia as both a dynamic center of floristic assembly and a long-term refuge for ancient taxa. This view is further supported by earlier phylogenetic studies that already hinted at shared biogeographic histories across Macaronesian and continental taxa. For instance, Vanderpoorten et al. [20], through a gain–loss analysis of synapomorphic pteridophyte species, identified a group of 31 taxa with common evolutionary origins distributed across the Azores, Madeira, and the Canary Islands, as well as in refugial areas of Europe and North Africa.

**Figure 1:**
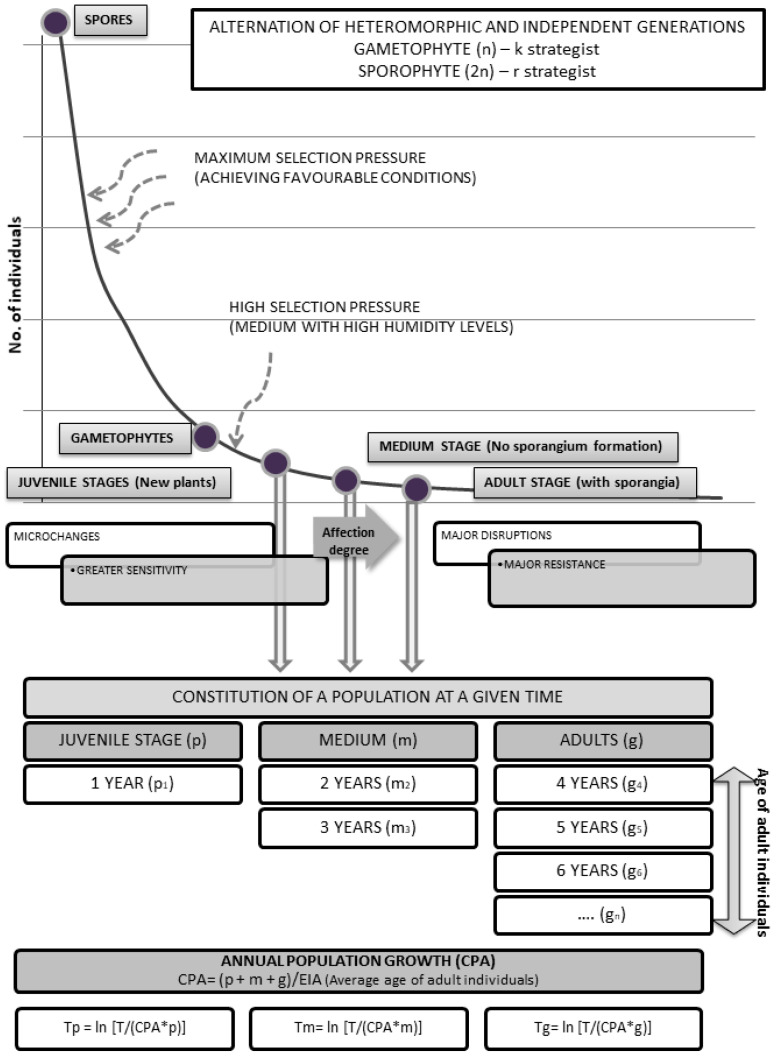
Graphical representation of the fern life cycle and the demographic dynamics of its populations.

Monilophytes, a diverse group of vascular plants, have evolved under selective pressures associated with increasingly arid continental environments [31]. Their life cycle is marked by an alternation of generations, in which the dominant sporophyte is vascularized, while the gametophytic generation is reduced to a thallophytic organism. This gametophytic stage is particularly sensitive to environmental conditions, as it requires consistently high-water availability for development and sexual reproduction. The need for an aqueous medium for fertilization, combined with its ecological independence from the sporophyte, represents a critical bottleneck subject to strong selective pressure (Figure 1). These physiological constraints largely confine monilophytes to humid and hyper-humid habitats. Of the approximately 12,000 recognized species, the vast majority are concentrated within the intertropical belt, where moisture conditions are favorable year-round. In contrast, species in extratropical regions tend to exhibit narrow distributions and are often associated with specific refugial habitats [32,33,34,35].

In certain extratropical regions, relicts of ancient palaeofloras can still be found, surviving under exceptional microclimatic conditions that mimic those of past warmer and more humid periods. These climatic refugia have allowed the persistence of pteridophyte lineages that once formed part of the Miocene flora, now largely extinct across continental landscapes. Notably, remnants of this ancient vegetation have been relegated to isolated redoubts within the Macaronesian archipelagos and the westernmost margins of the Mediterranean Basin. Among these, the territories surrounding the Strait of Gibraltar are of particular importance due to their singular topoclimatic conditions, which favor the survival of Neogene relicts [36]. Within this context, Pichi-Sermolli et al. [25] identify the Aljibic sector as a key pteridogeographical unit in the Iberian Peninsula, distinguished by its relictual fern flora, including *Culcita macrocarpa* C. Presl, *Diplazium caudatum* (Cav.) Jermy, and *Pteris incompleta* Cav. The ecological and biogeographical significance of these relictual ferns underscores the importance of assessing their current conservation status, particularly in regions that act as refugia. One such area is the mountain ranges near the Strait of Gibraltar, located within the Aljibic sector, which hosts several key populations of *C. macrocarpa, D. caudatum* and *P. incompleta*. This study aims to assess the demographic dynamics and climate sensitivity of the populations by applying empirical survival projections derived from annual abundance counts, alongside a principal component analysis (PCA) to identify key climatic drivers. We hypothesize that climate change significantly influences the survival of these relict ferns, while ongoing conservation programs have been effective measures supporting their persistence. By projecting the evolution of individual numbers over time and identifying environmental factors associated with population decline or persistence, this work seeks to contribute to the understanding of how microrefugial conditions and conservation efforts support the long-term survival of relict taxa under current and future climate scenarios.

## 2. Results

### 2.1. Correlation Between Climate and Demographic Data

To explore potential climate–abundance relationships at the local scale, pairwise Pearson correlation coefficients were calculated between the annual number of individuals in each population (Operational Geographical Unit, OGU) and standardized climatic variables for the period 2014–2023 (Table 1).

For *C. macrocarpa,* the overall correlations between climate variables and population abundance were weak to moderate across most OGUs. Several populations showed positive correlations with Tmax (e.g., OGU01: r = 0.44; OGU13: r = 0.39), while others showed negative or no relationships. The highest negative correlation was observed between DmaxHW and abundance in OGU02 (r = −0.69), suggesting a potential sensitivity to prolonged heat stress. ET was moderately to strongly negatively correlated in most OGUs, potentially reflecting moisture-related stress responses.

Populations of *P. incompleta* exhibited consistently strong and significant positive correlations with several climatic variables, particularly with DmaxHW (e.g., OGU23: r = 0.98; OGU25: r = 0.88), annual precipitation (e.g., OGU23: r = 0.86; OGU30: r = 0.86), and Tmax (e.g., OGU22: r = 0.84; OGU30: r = 0.87). These results suggest a general positive response of this species to warmer and wetter years with extended heat episodes, possibly reflecting a more opportunistic demographic behavior under fluctuating climate conditions.

For *D. caudatum*, the correlations revealed a more complex pattern. Several OGUs showed moderate negative correlations between Tmax and abundance (e.g., OGU34: r = −0.69), while others showed positive correlations (e.g., OGU33: r = 0.53). Notably, ET showed strong negative correlations in many populations (e.g., OGU36: r = −0.88; OGU44: r = −0.81), indicating a potential vulnerability to increased evaporative demand. DmaxCHD was generally positively associated with abundance, although the strength of these correlations was modest.

These explorations of correlations between abundance and climatic variables are further supported by the results of the Bayesian Hierarchical Model (BHM), whose details can be found in the Supplementary Materials. The effects and hyperparameters were appropriately estimated (Table S1). Tmax (Figure S1) and ET (Figure S5) exhibited the greatest variability among OGUs as varying slopes, highlighting differences in effects between populations. The effect of annual cumulative precipitation (Figure S3) showed an uncertain and inconsistent pattern across OGUs, similar to what was observed in Table 1. DmaxHW (Figure S4) and DmaxCHD (Figure S5) showed a systematic positive effect on abundance, a pattern also reflected in the mostly positive linear correlations. The hyperparameters for the varying intercepts across OGUs and for the temporal autocorrelation process displayed high standard deviation and autocorrelation values, indicating their importance in the model. This was further supported by improvements in the Watanabe–Akaike Information Criterion (WAIC) compared to simpler model structures.

### 2.2. Clustering in Operational Territorial Units (OTUs)

**Figure 2:**
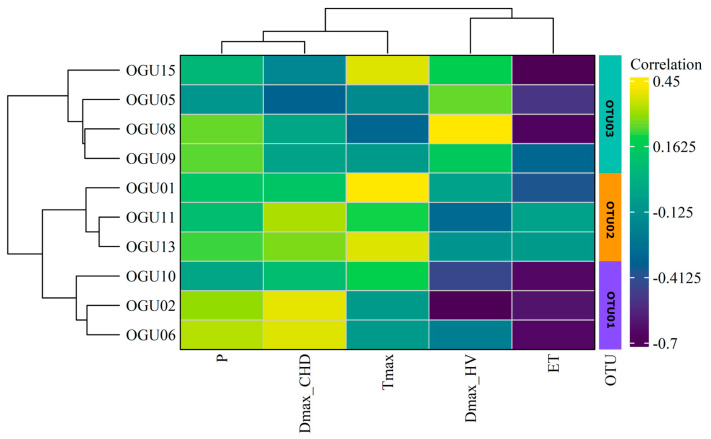
Hierarchical clustering dendrogram of OGUs for Culcita macrocarpa, grouped into OTUs based on similarities in their climate–abundance correlation profiles using Ward’s method. Each heatmap shows the correlation intensity between climatic variables and population abundance per OGU, with clustering of both OGUs (horizontal) and variables (vertical). Color gradients represent correlation strength. Climatic variables contributing most to group differentiation are shown on the right.

**Figure 3:**
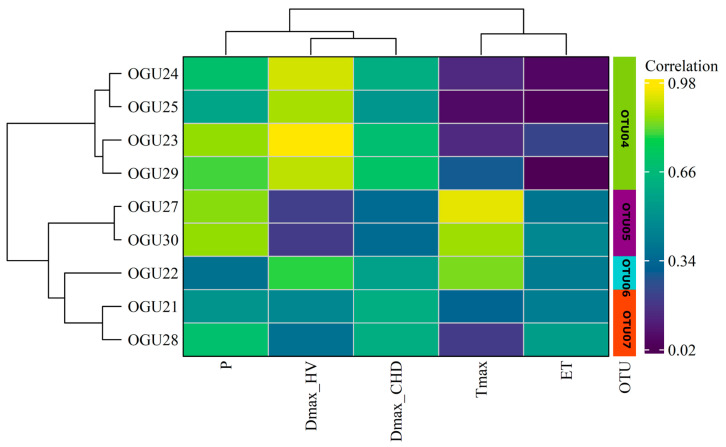
Hierarchical clustering dendrogram of OGUs for Pteris incompleta, grouped into OTUs based on similarities in their climate–abundance correlation profiles using Ward’s method. Each heatmap shows the correlation intensity between climatic variables and population abundance per OGU, with clustering of both OGUs (horizontal) and variables (vertical). Color gradients represent correlation strength. Climatic variables contributing most to group differentiation are shown on the right.

**Figure 4:**
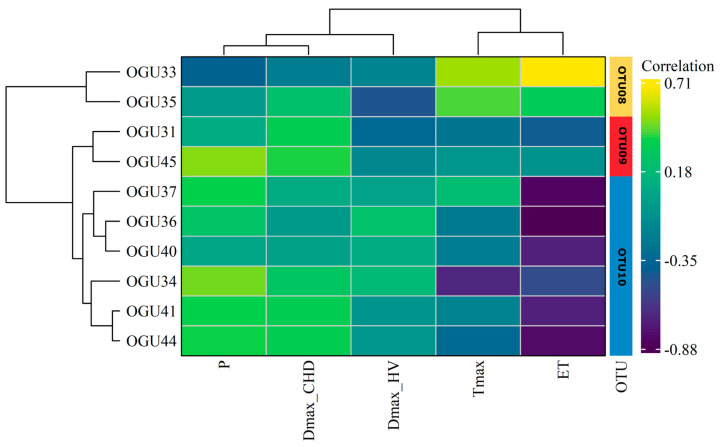
Hierarchical clustering dendrogram of OGUs for Diplazium caudatum, grouped into OTUs based on similarities in their climate–abundance correlation profiles using Ward’s method. Each heatmap shows the correlation intensity between climatic variables and population abundance per OGU, with clustering of both OGUs (horizontal) and variables (vertical). Color gradients represent correlation strength. Climatic variables contributing most to group differentiation are shown on the right.

To identify consistent patterns of climatic influence on population dynamics, a hierarchical clustering analysis was conducted using Ward’s method, based on the correlation profiles between annual species abundances and standardized climatic variables. The resulting dendrograms and heatmaps (Figure 2, Figure 3 and Figure 4) reveal distinct groupings of OGUs, which were subsequently categorized into OTUs to facilitate spatially structured interpretation. These OTUs represent populations with similar climate–abundance relationships. Numerical details can be consulted in Table 2.

For *C. macrocarpa* (Figure 2), three OTUs were identified. OTU01 includes OGUs 05, 08, 09, and 15, characterized by moderate positive correlations with the duration of heatwaves (DmaxHW) and weak or variable responses to other climatic factors. OTU02 groups OGUs 01, 11, and 13, which share strong positive associations with precipitation (P), maximum temperature (Tmax), and consecutive humid days (DmaxCHD), suggesting a greater dependence on humid and thermally stable environments. OTU03 includes OGUs 02, 06, and 10, defined by negative correlations with potential evapotranspiration (ET) and sensitivity to heat extremes (DmaxHW), reflecting possible stress under increased aridity.

For *P. incompleta* (Figure 3), four OTUs emerged. OTU04 comprises OGUs 23, 24, 25, and 29, which showed moderate to strong positive correlations with ET and Tmax, indicating better performance under warmer and drier conditions. OTU05 is represented solely by OGU26, which exhibited high sensitivity to a wide range of climatic variables (P, Tmax, DmaxCHD, DmaxHW), suggesting it may represent a unique ecological niche or transitional site. OTU06, consisting of OGUs 27 and 30, was primarily influenced by heatwave duration (DmaxHW), highlighting thermal extremes as the main climatic driver. OTU07 (OGUs 21, 22, and 28) was defined mainly by the number of consecutive humid days (DmaxCHD), emphasizing the importance of moisture continuity for this cluster.

For *D. caudatum* (Figure 4), three OTUs were delineated. OTU08 (OGUs 33 and 35) is associated with moderate negative correlations with ET and Tmax, indicating a preference for cooler and less evaporative conditions. OTU09 (OGUs 31 and 45) showed strong positive relationship to consecutive humid days (DmaxCHD), highlighting moisture as a critical factor for their abundance stability. OTU10, the largest group (OGUs 34, 36, 37, 40, 41, and 44), displayed strong negative correlations with ET and Tmax, and moderate positive responses to precipitation-related variables, indicating vulnerability to warming and drying trends.

### 2.3. Principal Component Analysis of Climate–Abundance Relationships

**Figure 5:**
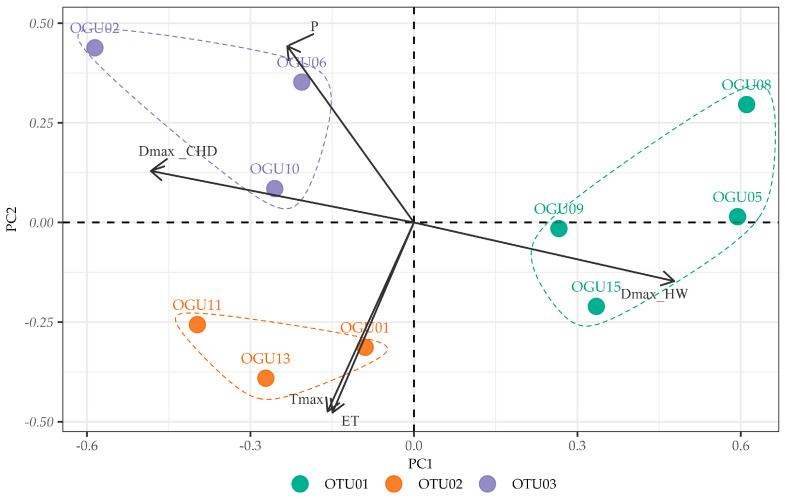
Principal Component Analysis (PCA) of the correlations between annual *Culcita macrocarpa* abundance and climate variables. The biplots show the distribution of Operational Geographical Units (OGUs) in the space defined by the first two principal components, which explain most of the total variance. Arrows indicate the contribution and direction of each climatic variable (Tmax = Yearly maximum monthly mean air temperature (°C); P = Annual cumulative precipitation (mm); DmaxHW = Maximum duration of heat waves (days); DmaxCHD = Maximum number of consecutive humid days (days); ET = Potential evapotranspiration (mm/month)). See Table 3 for the variable loadings.

**Figure 6:**
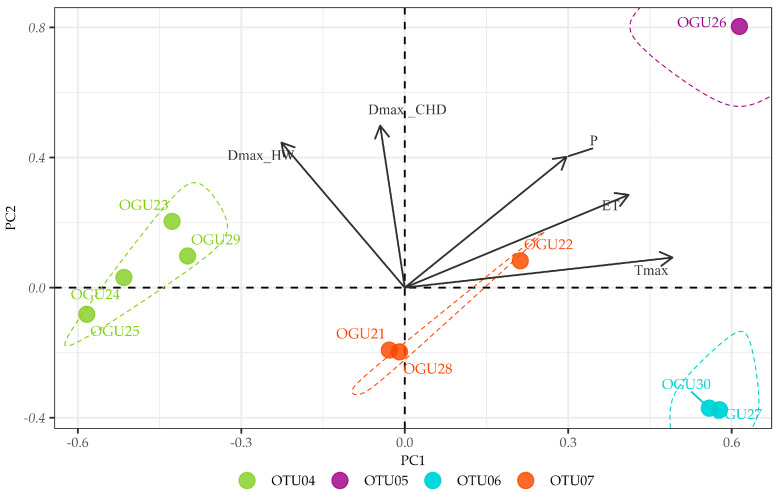
Principal Component Analysis (PCA) of the correlations between annual *Pteris incompleta* abundance and climate variables. The biplots show the distribution of Operational Geographical Units (OGUs) in the space defined by the first two principal components, which explain most of the total variance. Arrows indicate the contribution and direction of each climatic variable (Tmax = Yearly maximum monthly mean air temperature (°C); P = Annual cumulative precipitation (mm); DmaxHW = Maximum duration of heat waves (days); DmaxCHD = Maximum number of consecutive humid days (days); ET = Potential evapotranspiration (mm/month)). See Table 3 for the variable loadings.

**Figure 7:**
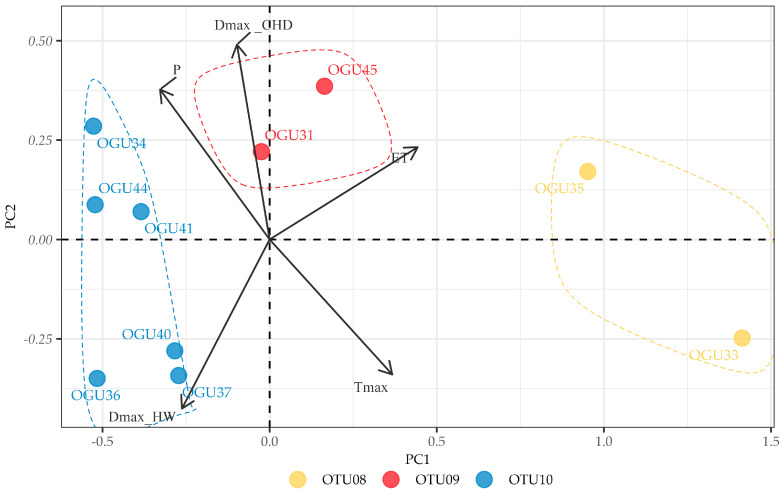
Principal Component Analysis (PCA) of the correlations between annual *Diplazium caudatum* abundance and climate variables. The biplots show the distribution of Operational Geographical Units (OGUs) in the space defined by the first two principal components, which explain most of the total variance. Arrows indicate the contribution and direction of each climatic variable (Tmax = Yearly maximum monthly mean air temperature (°C); P = Annual cumulative precipitation (mm); DmaxHW = Maximum duration of heat waves (days); DmaxCHD = Maximum number of consecutive humid days (days); ET = Potential evapotranspiration (mm/month)). See Table 3 for the variable loadings.

To examine the underlying structure of climate–species relationships and identify the main gradients of environmental variation, PCA was performed on the correlation matrices between species abundances and climatic variables for each species (Table 3; Figure 5, Figure 6 and Figure 7).

For *C. macrocarpa* (Figure 5), the first two principal components explained 77.43% of the total variance (PC1: 52.32%, PC2: 25.11%). PC1 was primarily defined by strong negative loadings of heatwave duration (DmaxHW = −0.79) and positive loadings of consecutive humid days (DmaxCHD = 0.52), representing a moisture–thermal stress gradient. PC2 was characterized by high positive loadings of maximum temperature (Tmax = 0.73) and evapotranspiration (ET = 0.58), indicating a thermal–evaporative demand axis.

For *P. incompleta* (Figure 6), the first two components accounted for 82.87% of variance (PC1: 54.19%, PC2: 28.68%). PC1 showed strong positive loadings for maximum temperature (Tmax = 0.76) and evapotranspiration (ET = 0.51), with moderate negative loading for heatwave duration (DmaxHW = −0.39), suggesting a thermal tolerance gradient. PC2 was dominated by heatwave duration (DmaxHW = 0.75) and consecutive humid days (DmaxCHD = 0.50), representing an extreme weather event axis.

For *D. caudatum* (Figure 7), 82.58% of variance was explained by the first two components (PC1: 57.41%, PC2: 25.17%). PC1 exhibited contrasting loadings between precipitation (P = 0.53) and temperature-related variables (Tmax = −0.46, ET = −0.50), defining a hydrothermal balance gradient. PC2 was characterized by opposing loadings of consecutive humid days (DmaxCHD = 0.71) and heatwave duration (DmaxHW = −0.66), representing a moisture stability-thermal stress axis.

### 2.4. Deterministic Population Projection Based on PCA-Derived Predictive Algorithm

Building on the multivariate structure revealed by the PCA, species-specific climate indices were developed by integrating variables with the highest absolute loadings (≥ |0.5|) on the first two principal components.

For *C. macrocarpa* (Equation (1)), the index incorporates the interaction between the maximum duration of consecutive humid days (DurmaxCHD) and maximum heatwave duration (DurmaxHW), as well as the product of maximum temperature (Tmax) and precipitation (P), weighted by heuristically adjusted coefficients. The resulting model predicts annual increases in abundance under conditions combining prolonged moisture and elevated temperatures with precipitation:

(1) $$N_{t}=N_{t-1}+13.24\cdot [1.57\cdot \left(DurmaxCHD\cdot DurmaxHW\right)+(Tmax\cdot P)]$$

In *P. incompleta* (Equation (2)), the algorithm emphasizes the combined effect of maximum temperature and evapotranspiration (ET), along with the interaction between heatwave duration and consecutive humid days. The model predicts population declines under scenarios of high temperature and hydric stress:

(2) $$N_{t}=N_{t-1}-10.75\cdot [\left(2\cdot Tmax\cdot ET\right)+(DurmaxHW\cdot DurmaxCHD)]$$

For *D. caudatum* (Equation (3)), the index integrates the negative interaction between precipitation and evapotranspiration, as well as the combined effect of heatwave duration and consecutive humid days, reflecting the species’ sensitivity to water low availability and thermal extremes:

(3) $$N_{t}=N_{t-1}-11.79\cdot \left[-0.79\cdot P\cdot ET-\left(DurmaxHW\cdot DurmaxCHD\right)\right]$$

The heuristic demographic models produced abundance trajectories that broadly mirrored the empirical trends for each species, supporting their utility for exploring climate-driven population responses. The comparison between observed and modeled individual counts from 2016 to 2022 (Table 4 and Table 5) demonstrates a generally satisfactory concordance, with positive differences (Observed > Modeled) in most years indicating effective conservation outcomes, while negative differences highlight periods where climatic impacts were not fully buffered.

Model performance varied across species, with *C. macrocarpa* showing the lowest prediction errors and thus the highest agreement between observed and modeled values (MAE: 10.29; RMSE: 13.54; MBE: 8.29; MAPE: 2.78%) (Table 4 and Table 5). In contrast, *P. incompleta* exhibited the highest error metrics (MAE: 59.29; RMSE: 73.50; MBE: 24.71; MAPE: 11.80%), indicating lower accuracy in reproducing observed population trends. *D. caudatum* showed intermediate performance, with a MAE of 43.57, RMSE of 70.40, MBE of 27.32, and MAPE of 11.10%. These results reflect notable differences in model fit among the species, with *C. macrocarpa* performing best and *P. incompleta* the least accurately predicted.

### 2.5. Comparison of Population Projections Derived from Empirical and Climate-Driven Models

Model performance was evaluated by comparing projected and observed population sizes from 2016 to 2022 using four standard metrics calculated from 100,000 bootstrap simulations (Table 6). *C. macrocarpa* showed the lowest errors, with a mean absolute error (MAE) of 46.63 ± 26.67 individuals, root mean square error (RMSE) of 56.70 ± 32.69, mean bias error (MBE) of −40.14 ± 35.92, and mean absolute percentage error (MAPE) of 12.95 ± 8.18%. *D. caudatum* and *P. incompleta* exhibited substantially higher errors: *D. caudatum* had MAE of 282.25 ± 30.18, RMSE of 326.79 ± 32.48, MBE of −281.38 ± 31.08, and MAPE of 52.84 ± 5.88%; *P. incompleta* showed MAE of 188.68 ± 12.60, RMSE of 212.82 ± 13.64, MBE of −188.63 ± 12.65, and MAPE of 47.92 ± 3.31%. In all cases, the bias was negative, indicating systematic underestimation of population sizes by the models.

**Figure 8:**
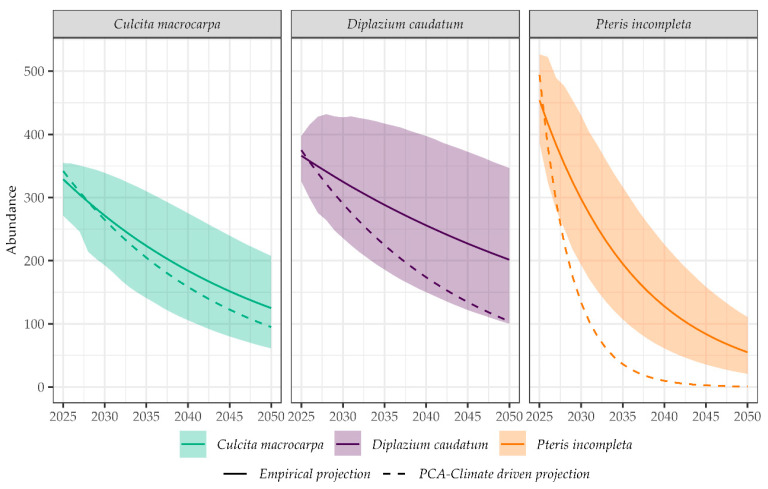
Comparison of population trajectories from 2025 onwards according to deterministic empirical projections and climate-driven projections. Shaded areas represent 95% confidence intervals for empirical projections.

Population projections from empirical deterministic models, based on observed annual population change rates (2014–2022) and assuming stationary environmental conditions, were compared with climate-driven projections under the RCP4.5 scenario (Figure 8). All species showed declining trends through 2050 in both modeling approaches, but with differences in magnitude and trajectory. Climate-driven projections under the Representative Concentration Pathway 4.5 (RCP4.5) scenario consistently forecast more severe population declines for all three species compared to empirical projections. For *C. macrocarpa*, the climate-driven model estimated a population size of approximately 95 individuals by 2050, whereas the empirical projection predicted a mean of 125 individuals (95% CI: (61, 208)), indicating a 24% greater decline under the climate-based scenario (95% CI: (55%, 54%)). In the case of *D. caudatum*, the climate-driven projection estimated around 104 individuals, compared to an empirical estimate of 45 (28, 68) individuals. This suggests that the climate-driven model predicts a substantially smaller decline, approximately 56% (34%, 73%) less, compared to the empirical projection. For *P. incompleta*, the discrepancy between models was most pronounced: the empirical model projected a mean of 11 (3, 27) individuals, whereas the climate-driven model forecasted a sharp reduction to a single individual, corresponding to a 90% (66%, 96%) greater decline under the climate scenario.

## 3. Discussion

Current anthropogenic climate change [37] poses a major threat to sensitive ecosystems such as the Alboran Arc, which serves as a refugium for multiple relict fern species from the Paleomediterranean flora. Increasing rates of population extinction have been directly linked to the effects of climate change, with the most vulnerable species typically being stenoecious taxa restricted to exceptional topoclimatic conditions [1,38,39]. Recent evidence identifies the Mediterranean Basin as a climate change hotspot, where rising temperatures and an increased frequency of extreme events are expected to drive substantial biodiversity loss and widespread habitat contraction [40]. The vulnerability of Mediterranean flora is particularly pronounced among endemic and relict species, many of which exhibit narrow ecological niches and limited dispersal capacities, rendering them highly susceptible to both direct and indirect effects of climate change [40]. Within this context, the long-term persistence of relict ferns in the Alboran Arc will likely depend not only on their physiological tolerance to increasing temperatures and drought, but also on the effectiveness of targeted conservation actions and the preservation of microrefugia within protected areas. These microrefugia are often associated with forest ecosystems (“canutos”), which act as key habitat-forming structures for these fern species [41], yet are themselves predicted to be vulnerable to future climatic shifts [42].

Our results demonstrate that climate change is already altering the viability of relict fern populations in the Alboran Arc, particularly those adapted to historically warm and humid microclimates. Among the three species examined, *P. incompleta* exhibited the greatest sensitivity, with projected declines closely associated with rising maximum temperatures and the increasing duration of heatwave periods. These local patterns reflect broader global trends, where ferns are recognized as highly vulnerable to climate change due to their physiological dependence on stable moisture and temperature regimes and emerging evidence suggests that suitable habitats for many fern species are shrinking globally, particularly for taxa with narrow ecological niches or limited dispersal ability. Shifts in fern richness are expected geographically and altitudinally globally. According to Huang et al. [43], diversity in ferns will remain concentrated in low-latitude regions but increasingly accumulate at higher elevations, where more favorable moisture conditions persist. Projections for tree ferns in the Atlantic Forest indicate that most species will lose most of their habitat by 2050, with some facing high extinction risks [44]. Similarly, Wang et al. [45] highlighted climate-driven range contractions for several endangered species, underscoring the urgency of integrating adaptive strategies into conservation planning. Umair et al. [46] support this pattern, showing that on the Tibetan Plateau fern richness peaks at mid-elevations with optimal moisture indices, while richness declines at both lower and higher extremes due to heat stress and excessive solar radiation, respectively. Moreover, recent experimental and modeling studies have provided direct evidence that future climate scenarios, especially those involving higher emissions, will dramatically increase the extinction risk for a wide range of plant taxa, including ferns. For instance, Hollenbeck and Sax [47] found that under moderately high emission scenarios, 5–36% of tropical montane epiphyte species may face extinction by the end of the century, with the risk being particularly acute for species confined to narrow elevational bands or microrefugia. This pattern is consistent with our results, which highlight the acute vulnerability of *P. incompleta* to projected increases in temperature extremes and the frequency of climatic anomalies in the Alboran Arc.

Analysis of fern population responses to climate during the past decade revealed consistent clustering patterns based on shared climatic sensitivity, highlighting that populations tend to group according to similar responses to environmental drivers. Notably, our results also underscore that populations belonging to the same species can display divergent climate–abundance relationships depending on local conditions, reinforcing the importance of fine-scale ecological context in shaping population trajectories. These OTU-based groupings offer a valuable framework for future spatially explicit modeling and conservation prioritization. However, their effective application will require the integration of local-scale factors, which often remain inaccessible or unmeasured in conventional monitoring efforts. Microclimatic variability, driven by topographic complexity, vegetation structure, and soil heterogeneity, can generate substantial environmental differences over very short distances [48], yet such heterogeneity is typically lost in coarse-resolution climatic datasets. As a result, species tightly linked to microsite conditions may be misrepresented in large-scale distribution models, potentially leading to erroneous predictions regarding habitat suitability and persistence. Moreover, local abundance is not solely governed by macroclimatic variables but is also influenced by a suite of fine-scale ecological processes, including disturbance regimes and successional dynamics [49], biotic interactions [50], dispersal limitations and landscape fragmentation [51], as well as stochastic demographic and environmental factors [52]. The integration of these local drivers is essential to accurately assess population resilience and to inform conservation actions that go beyond climate projections.

The comparison between empirically derived and climate-driven projections reveals substantial differences in both predictive accuracy and projected population trajectories for the three studied fern species. In all species, the empirical model predicts smoother and less drastic declines in abundance than climate driven. The empirical model, which demonstrated the lowest error metrics for *C. macrocarpa*, suggests that this species’ population dynamics during the observation period were well approximated by stationary processes, likely due to either reduced sensitivity to interannual climate variability or greater demographic inertia. This finding is consistent with previous work demonstrating that some fern species, especially those with stable habitats, can exhibit relatively stable population trends under current conditions [53].

In contrast, projections for *D. caudatum* and *P. incompleta* exhibited greater discrepancies, both in their deviation from observed population data and in the divergence between empirical and climate-driven trends. Notably, both modeling approaches systematically underestimated population sizes, as evidenced by consistently negative MBE. While the climate-driven models yielded lower overall prediction errors compared to the empirical models, their outputs still failed to capture recent increases in population abundance, likely due to the omission of local-scale reinforcement actions and microenvironmental factors not represented in coarse-resolution climate datasets [54,55].

The contrasting projections of population trajectories under the climate-driven and empirical models reveal important insights into the potential impacts of climate change on the studied species. Notably, the climate-driven projections consistently forecasted more severe declines for *C. macrocarpa* and *P. incompleta*, while predicting a comparatively less pronounced decline for *D. caudatum. Pteris incomplea* exhibits an important divergence in projections between models. Climate-driven models anticipate a sharp population collapse by mid-century, underscoring the species’ heightened vulnerability to projected increases in temperature and the frequency of extreme climatic events. While such forecasts may reflect genuine climate sensitivity, they also amplify the uncertainty associated with extrapolating climate-demographic relationships beyond the observed range. The confidence intervals surrounding the empirical projections, estimated via 100,000 bootstrap replicates, capture a broader range of potential outcomes, offering a more precautionary representation of demographic uncertainty.

The systematic underestimation observed in the empirical model projections, reflected by consistently negative MBE, is primarily attributable to the exclusion of population reinforcement activities conducted during the study period. These interventions involved the periodic introduction of cultivated individuals into wild populations, which were not accounted for in the deterministic models designed to simulate natural demographic processes alone. Specifically, 46 individuals of *C. macrocarpa* were introduced into a single population in 2015; *D. caudatum* received 325 individuals across four populations between 2018 and 2022 over eight separate events; and for *P. incompleta*, although exact numbers per reinforcement are unspecified, substantial introductions occurred in two populations during 2015 and 2018, with a total of 163 and 234 individuals, respectively. Consequently, when model performance was assessed by comparing projected versus observed population sizes over the past decade, the omission of these anthropogenic reinforcements resulted in systematic underestimation and notable discrepancies between predicted and actual abundances. It is important to emphasize that this modeling approach was intentional, as the objective was to estimate how population abundances would evolve in the absence of reinforcement actions. This provides a valuable counterfactual baseline, allowing us to identify the intrinsic vulnerabilities of each species.

These findings underscore the effectiveness of reinforcement measures in shaping population trajectories and highlight the limitations of models that omit such interventions when used for retrospective evaluation. Considering both modeling approaches, it becomes evident that in the absence of conservation and reintroduction actions, particularly those applied to *D. caudatum* and *P. incompleta*, the models would more accurately reflect the actual vulnerability of these populations, thereby demonstrating the success of these measures. However, the pronounced discrepancies between empirical and climate-driven projections raise concerns about relying solely on either framework for conservation planning. Instead, integrating both perspectives offers a more nuanced and precautionary outlook, especially for species with high ecological sensitivity or restricted distribution ranges. Both models remain highly valuable for conservation planning, as they help identify critical thresholds below which populations are likely to decline without continued management. To enhance the predictive accuracy and ecological realism of future projections, it would be advisable to develop modeling frameworks that explicitly incorporate the timing and magnitude of reinforcement actions. This would allow for more refined evaluations of alternative management scenarios. Nevertheless, the current approach yields essential insights into the underlying demographic risks and highlights the potential accumulation of “conservation debt” should reinforcement efforts be reduced or discontinued. Given the substantial model divergences observed here, conservation strategies should remain flexible and supported by long-term monitoring programs that allow projections to be validated and management actions to be adjusted as new empirical data emerge under changing environmental conditions.

Although the projection models yielded satisfactory results in terms of performance, particularly with respect to MAPE, and addressed the need to evaluate whether conservation programs contribute to the maintenance of the three relict paleomediterranean fern populations, the proposed models exhibit several limitations that warrant further discussion.

(1) Incorporating PCA-derived climatic variable weights into the population projection algorithm constitutes a multivariate approach to link environmental variability with demographic trends in relict fern populations. This framework allows for the simultaneous consideration of key climatic drivers and their combined influence on population trajectories. However, this methodology has inherent limitations. The model’s linear and additive structure may oversimplify complex biological processes that are often non-linear and influenced by threshold effects, feedback mechanisms, or interactions among climatic and ecological factors. Furthermore, it assumes that the principal components captured by PC1 and PC2 fully represent the climatic drivers of population change, potentially neglecting other important abiotic or biotic influences. Critical demographic processes such as reproduction, mortality, dispersal, and genetic variability are also absent from this model, limiting its ability to fully capture population viability.
(2) The deterministic survival projection model, grounded in empirical annual rates derived from a decade of census data, offers a practical tool for projecting population trends. By capturing the net effects of demographic and environmental factors observed during the study period, the model reflects the integrated trajectory of population change. However, its assumption of temporal constancy in demographic conditions, given that the bootstrap simulations are based solely on past observed rates, limits its reliability under dynamic scenarios, such as those induced by climate change, land-use transformations, or unforeseen ecological disturbances. The model does not account for demographic stochasticity, density dependence, interannual variability, or the impact of rare but consequential events like extreme droughts or disease outbreaks, all of which can be particularly influential in small, isolated populations.
(3) The divergence between observed and modeled population abundances has been used as indicator of conservation effectiveness. While this approach offers valuable preliminary insights, it is important to acknowledge that such differences may also arise from a range of other ecological and demographic dynamics not fully captured in the current modeling framework. These include natural population variability and external environmental factors that are inherently challenging to parameterize comprehensively. Additionally, model–data mismatch is an expected outcome when dealing with complex ecological systems. As such, while the observed–predicted discrepancies can help identify potential conservation outcomes, they should be interpreted within the broader context of model assumptions, data limitations, and ecological variability.

These limitations highlight the necessity for future research to adopt more flexible and advanced modeling frameworks. Approaches such as Bayesian hierarchical models and other non-linear methodologies could better accommodate the complex ecological responses and interactions characteristic of relict fern populations, ultimately enabling more accurate predictions of their long-term viability under accelerating environmental change. Despite its limitations, the PCA-derived projection model remains a valuable tool for preliminary climate impact assessments and supports adaptive conservation strategies. Meanwhile, the deterministic model provides a crucial baseline for evaluating population trends and determining the need for intervention. Used together, these models offer complementary insights, helping differentiate between intrinsic demographic changes and climate-driven influences. Although heuristic and not formally optimized, their performance metrics demonstrate satisfactory predictive concordance for hypothesis generation. The results indicate that empirical models based on stationary conditions provide important insights into the intrinsic demographic dynamics of these threatened ferns, while integrating climate projections reveals a more pessimistic outlook for population viability. The pronounced discrepancies between observed and projected values further underscore the critical role of reinforcement activities in sustaining these populations and highlight the need for future modeling approaches that explicitly incorporate both natural demographic processes and targeted conservation interventions. Collectively, these insights reinforce the conclusion that fern distributions are strongly governed by climate, and that their future persistence will depend on a combination of intrinsic tolerance, the availability of climatic refugia, and proactive conservation measures designed to buffer populations against increasing environmental extremes.

## 4. Materials and Methods

### 4.1. Study Area

**Figure 9:**
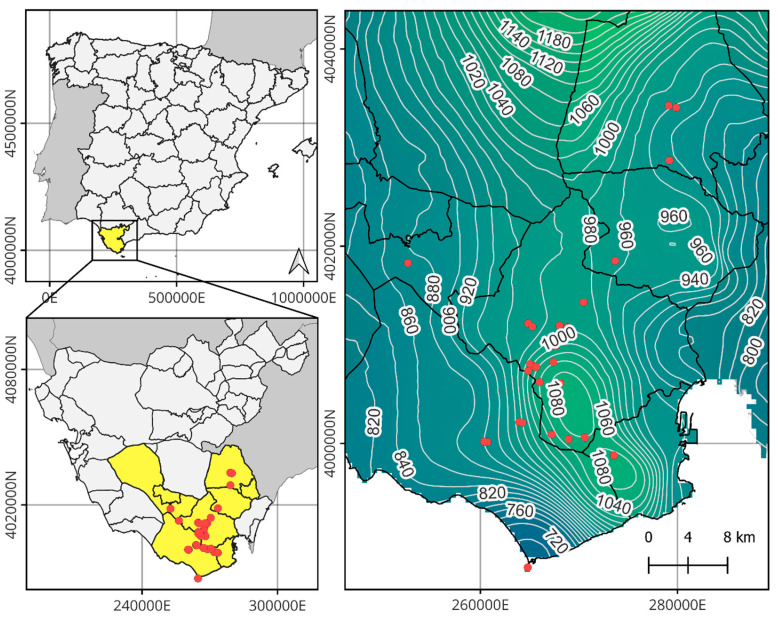
Geographic location of the study area (mountain ranges near the Strait of Gibraltar). The map displays isohyets of mean annual precipitation for the period 2011–2020 [62]. Dots indicate the centroids of mapped populations of *Culcita macrocarpa*, *Diplazium caudatum*, and *Pteris incompleta*, which are monitored annually as part of the Andalusian Fern Recovery Plan. Although all known populations are shown, this study analyzed only 10 populations per species, due to limited access to tabular data on individual counts rather than precise spatial coordinates.

The study area is located in the mountain ranges near the Strait of Gibraltar, in the southern part of Cádiz province (Spain) (Figure 9). These ranges form part of the Aljibic sector and are predominantly composed of the Campo de Gibraltar Flysch, characterized by quartzitic sandstones and generally nutrient-poor soils. Elevations range from 340 m, where the fog belt begins, to a maximum of 1091 m at Pico del Aljibe, with Pico de Luna (786 m) standing as the highest summit in the immediate surroundings of the Strait. These peaks are frequently shrouded in dense fog, generated by humid Atlantic and Mediterranean winds, resulting in significant cryptoprecipitation that sustains a distinctive and highly specialized flora [56]. The region experiences moderate thermal variation, with average temperatures ranging from 7 °C to 27 °C and a mean annual temperature of approximately 15.7 °C. Precipitation is markedly seasonal, averaging 1065 mm annually and exceeding 1300 mm in some zones [57].

From a bioclimatic perspective, the study area belongs to the thermo-Mediterranean belt, with an ombroclimate ranging from upper sub-humid to hyper-humid. Biogeographically, it is part of the Aljibic Sector within the coastal Lusitanian-Andalusian Province of the Mediterranean Region [58]. According to Rivas-Martínez et al. [59], the potential vegetation (climax) corresponds to cork oak forests with wild olive in the lower elevations (*Oleo-Quercetum suberis*) and to Andalusian gall oak woodlands (*Rusco hypophylli-Quercetum canariensis*) in summit zones. Particularly noteworthy are the edaphophilic communities found in narrow ravines with abundant leaf litter, where the *Frangulo-Rhododendretum baetici* series develops. Of special interest are two plant communities that host the relict ferns studied in this work. First, *Diplazio caudati–Ocoteetum foetentis*, a lauriphyllous forest association analogous to those in Macaronesia, although less defined in the mainland context. This community represents the mature stage of humid thermo- to meso-Mediterranean edapho-hygrophilous series, typically located in areas with persistent fog, high cryptoprecipitation, or along watercourses and ravines [60]. It is dominated by *D. caudatum*, with occasional occurrences of *P. incompleta* under the most humid microconditions. Second, *Scrophulario laxiflorae–Rhododendretum baetici* is a tall, lauroid shrub community confined to stream heads and gorges with sustained environmental humidity [61]. This dense formation is composed of evergreen, sclerophyllous nanophanerophytes such as *Rhododendron ponticum*, *Frangula alnus* subsp. *baetica*, *Laurus nobilis*, *Ilex aquifolium*, and *Hedera canariensis*, with a characteristic fern layer dominated by *Athyrium filix-femina*, *Blechnum spicant*, *Osmunda regalis*, and, in exceptionally humid niches, *C. macrocarpa*.

### 4.2. Ecology of the Studied Species and Key Threats

*Culcita macrocarpa*, *D. caudatum* and *P. incompleta*, as paleomediterranean relict species that find refuge in the study area have been analyzed (Table 7). For each, we present the currently accepted scientific name (based on Gbif.org and POWO, 2024), their family classification following the PPG I system (Pteridophyte Phylogeny Group classification of the ferns and lycophytes) [63], and their geographical distribution. We also include the number of species of the same genus in Europe and the broader Mediterranean region relative to the global total, as a proxy for biogeographic relictualism. Additional traits relevant to their evolutionary history are considered, such as chromosome number, which is notably high in all three species, and the type of laesura on their spores (either trilete or monolete). Chromosome number and genome size are recognized as informative cytogenetic characters for assessing evolutionary age and diversification in monilophytes, due to their strong phylogenetic signal and relevance for taxonomic inference across fern lineages [64,65,66]. Likewise, spore laesura morphology, trilete (Y-shaped) or monolete (linear), reflects different evolutionary trajectories, with trilete forms generally interpreted as ancestral and monolete as derived [67]. Finally, the various legal and conservation frameworks concerning the protection status of the studied species are outlined. Notably, none of them are included in the IUCN Red List of Threatened Pteridophytes in Europe [68]. This omission stems from the fact that the assessment adopts a strictly administrative definition of continental Europe, thereby incorporating the Macaronesian archipelagos of Spain and Portugal.

*Culcita macrocarpa, D. caudatum* and *P. incompleta* persistence is restricted to microhabitats with topoclimatic conditions that closely mirror the bioclimatic environments of their ancestral ranges [15]. The three species exhibit marked ecological convergence, occupying a highly specialized niche characterized by pronounced sciophily and hygrophily. These ferns are confined to humid, thermally buffered forest understories, typically ranging from 15 °C to 30 °C, with minimal annual temperature variation [15,69,70]. Their distributions are closely tied to specific microhabitats such as *canutos*, narrow, north-facing ravines, shaded streambanks, and deeply incised valleys, where persistent fog, dense canopy cover, and perennial water availability ensure stable moisture regimes year-round [36,41]. Edaphic factors also play a crucial role: while *C. macrocarpa* tolerates a broad range of substrates except calcareous soils, and is found in both deep and skeletal profiles, *P. incompleta* favors highly acidic, organic-rich soils, and *D. caudatum* shows a strong affinity for siliceous substrates [60]. All three species exhibit heightened sensitivity to microclimatic disturbance, particularly during spore germination and early gametophyte development [29].

The persistence of *C. macrocarpa*, *D. caudatum*, and *P. incompleta* is critically dependent on microhabitats that maintain high humidity and stable thermal regimes, conditions increasingly threatened by a range of environmental pressures. These include both global-scale drivers such as climate change and more localized anthropogenic disturbances [69,71,72].

**Figure 10:**
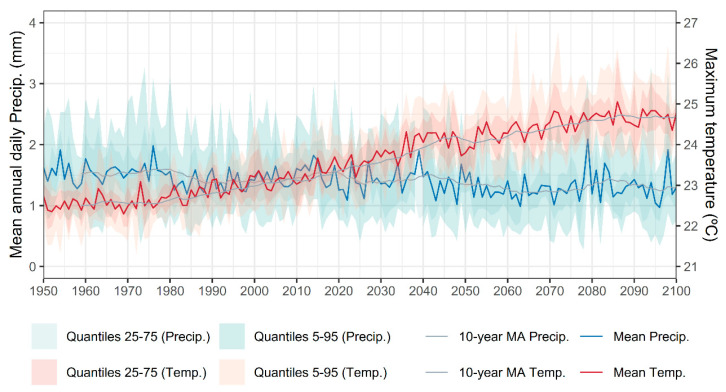
Mean annual maximum temperature and mean annual daily precipitation for the study area from 1950 to 2100. The figure shows both the annual mean values and 10-year moving average trend lines, along with 75% and 95% credibility intervals. The ensemble mean corresponds to the average of CMIP6 climate models available through AdapteCCa [73]. Credibility intervals were calculated using quantiles based on the 46 available models.

**Figure 11:**
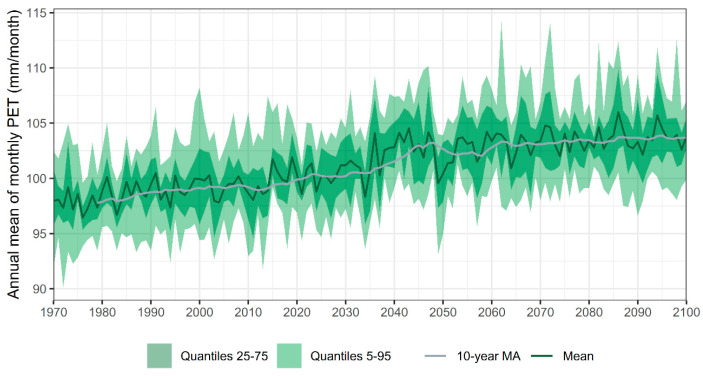
Annual mean of monthly Potential Evapotranspiration (PET) for the study area from 1950 to 2100. The figure shows both the annual mean values and 10-year moving average trend lines, along with 75% and 95% credibility intervals. The ensemble mean corresponds to the average of CMIP6 climate models available through AdapteCCa [73]. Credibility intervals were calculated using quantiles based on the 46 available models.

(1) Habitat degradation. The conversion of native laurel and mixed forests to monoculture plantations, particularly *Eucalyptus spp*., leads to soil desiccation and loss of shaded, humid niches. Wildfires, especially prevalent in mainland Portugal, further threaten *C. macrocarpa*, already classified as Critically Endangered in that region. Similarly, *D. caudatum* and *P. incompleta* are highly sensitive to the degradation of hygrophilous forest ravines and streambanks where they occur.
(2) Hydrological alterations, such as water extraction, river channeling, and wetland desiccation, directly impact the moisture-dependent habitats of these species. Contamination from agricultural runoff, livestock waste, and untreated urban or industrial effluents further degrades water quality, altering edaphic and microclimatic conditions essential for fern survival.
(3) Land-use changes, including deforestation, land clearing for agriculture, and infrastructure development (e.g., roads, firebreaks), fragment habitats and reduce the extent of suitable environments. Overgrazing contributes to trampling, herbivory, and soil nitrification, which in turn promotes colonization by competitive native or invasive species, displacing native ferns.
(4) Recreational pressures, such as unregulated tourism, hiking, and the construction of leisure infrastructure, can result in physical disturbance to sensitive fern populations, especially when located near trails or accessible forested areas.
(5) Population isolation and small population sizes exacerbate genetic erosion and vulnerability to stochastic events, including droughts, landslides, and fires. The scattered and fragmented nature of existing populations limits gene flow and reduces resilience.
(6) Biotic threats, such as invasive plant species, increase competition for light, space, and moisture. Although specific pathogens or pests have not yet been documented in these species, their potential impact remains a concern, particularly under changing environmental conditions.
(7) Climate change emerges as a major overarching threat. Projections under the RCP 4.5 scenario [73] indicate a sustained increase in mean annual maximum temperatures and a decline in annual precipitation (Figure 10), leading to higher evapotranspiration rates and, consequently, reduced water availability (Figure 11). These changes are particularly detrimental during the most sensitive stages of the fern life cycle, spore germination, gametophyte growth, and fertilization, where moisture is essential for success.

In summary, the interplay between climate-driven changes and anthropogenic habitat disturbance represents a multifaceted threat to the survival of these relict ferns. Their narrow ecological tolerances, low dispersal capacity, and reproductive constraints make them highly vulnerable to even subtle shifts in habitat quality and availability. In this context, assessing their responses to climatic variables, the most readily measurable stressors, plays a key role in predicting the future viability of these species within the study area.

### 4.3. Methods

**Figure 12:**
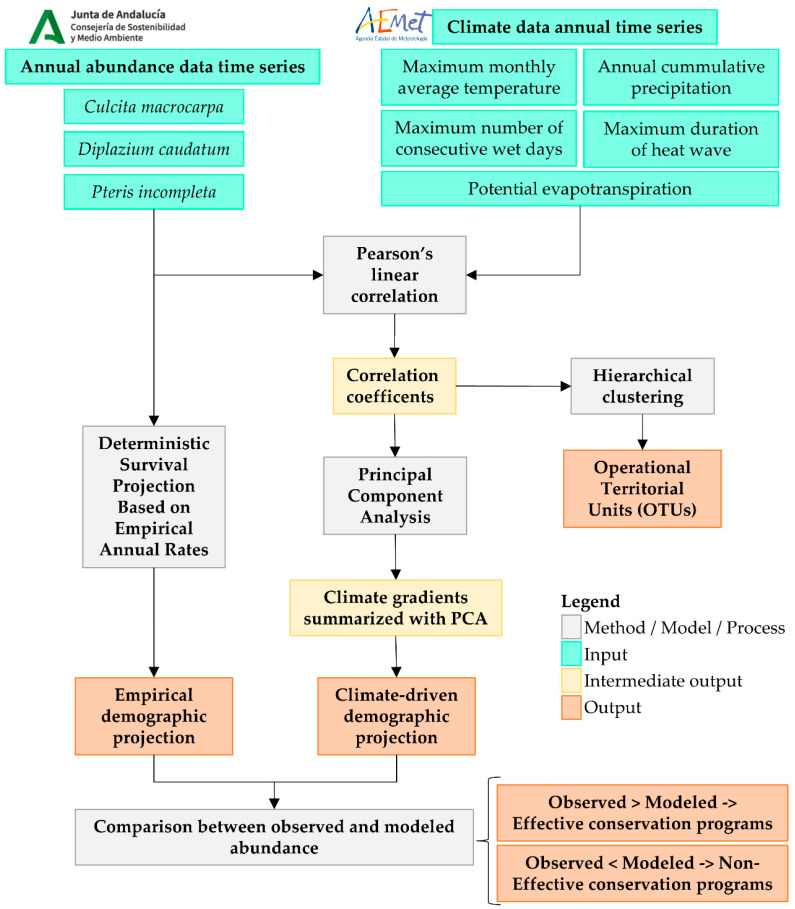
Graphical representation of the workflow developed in this study. The diagram outlines the sequential steps from data collection and processing through statistical analyses (including correlation analysis, cluster analysis, and PCA) to demographic projections based on empirical annual survival rates and climatic variables.

The graphical representation of the workflow employed in this study is shown in Figure 12.

#### 4.3.1. Structured Abundance Data of Fern Populations

Abundance data for *C. macrocarpa*, *D. caudatum*, and *P. incompleta* were obtained from restricted-access records provided by the Andalusian Fern Recovery Plan [71]. This program systematically delineated known populations of the target species within Alcornocales Natural Park. Since 2014, the number of individuals in each population has been recorded annually, with consistent sampling areas maintained over time, an important aspect that enhances the dataset’s reliability for population trend analysis.

The dataset comprises 1037 records, corresponding to combinations of monitored populations and years. In total, 50 distinct populations were surveyed: 22 for *C. macrocarpa*, 17 for *D. caudatum*, and 11 for *P. incompleta*. However, data completeness varies across populations, with missing values ranging from 7.14% to 81.8%, and a median missing rate of 27.2%. The first and third quartiles are 16.8% and 42.1%, respectively. At the species level, the proportion of missing data is 18.4% for *P. incompleta*, 26.2% for *C. macrocarpa*, and 21.1% for *D. caudatum*. Sampled population areas range from 0.11 to 3.81 hectares, with a median of 0.68 ha. Notably, populations of *C. macrocarpa* and *D. caudatum* tend to occupy smaller areas than those of *P. incompleta*, with observed area ranges of [0.24, 1.15], [0.34, 1.26], and [0.65, 2.60] ha, respectively.

Due to confidentiality restrictions on access to conservation data, only 10 populations per species, referred to here as Operational Geographical Units (OGUs), were available for analysis. These OGUs were selected from the broader dataset based on data accessibility and the continuity of abundance records. Despite the limited subset, these populations provide representative coverage across the environmental gradient of Alcornocales Natural Park, and were therefore used as the fundamental units for subsequent climatic and demographic modeling.

#### 4.3.2. Climate Data Description

The selected climatic variables (Table 8) were chosen for their expected influence on critical stages of the biological cycle of relict fern species, particularly spore germination, gametophyte development, and overall population viability, under Mediterranean climate conditions. Climatic data were obtained from the *San Roque* meteorological station (Cádiz), operated by AEMET [74]. The station is located at 36°16′23″N, 5°17′4″W, at an altitude of 1 m above sea level, and approximately 20 km from the fern populations within Los Alcornocales Natural Park. Projections were obtained through the AdapteCCa Climate Change Scenario Viewer [73], under the RCP 4.5 scenario. The data correspond to the ensemble mean of all available climate models from the CMIP6 (Coupled Model Intercomparison Project Phase 6) framework included in the platform. Variables were standardized as z-scores (mean-centered and scaled to unit variance).

#### 4.3.3. Correlation Analysis and Grouping in Operational Territorial Units (OTUs)

A correlation analysis was performed using P.A.S.T. software (v. 4.17c) [75] to assess the relationship between abundance of individuals for each species in each Operational Geographic Unit (OGU, understood as a population unit), and local climatic variables. Specifically, Pearson’s linear correlation coefficients (r) were computed for each species abundance–climate variable pair across OGUs.

Subsequently, the resulting correlation matrices were subjected to hierarchical cluster analysis using Ward’s method to identify patterns among OGUs. This analysis produced dendrograms reflecting the similarity structure of the OGUs, which were then used to delineate broader Operational Territorial Units (OTUs) based on shared climatic-abundance profiles. OTUs aggregate OGUs exhibiting comparable relationships between species abundance and climatic variables, thereby reducing spatial complexity and facilitating the identification of ecologically coherent regions. This approach reveals regional clusters of species population responses to climate, enhances the robustness of subsequent modeling by grouping areas with similar climatic sensitivities, and provides practical insights for conservation planning by defining climate-relevant management units. Overall, OTUs offer a valuable framework to integrate fine-scale ecological data with larger-scale spatial and climatic patterns.

As a complementary analysis, a Bayesian Hierarchical Model was developed to assess the variability in the effects of climatic variables on individual abundance across the different OGUs. The model included fixed effects, varying intercepts by OGU, varying slopes by OGU, and a first-order autoregressive (AR(1)) process as random effects to capture both the hierarchical structure and temporal autocorrelation in the data. Further details on its implementation can be found in the Supplementary Material, specifically in Equation (S1).

To identify the most relevant climatic variables for each species, a Principal Component Analysis (PCA) was conducted independently for each species. Although the species share broadly similar ecological niches, they exhibit distinct habitat preferences (as detailed in the Species Ecology section), justifying a species-specific approach to capture potential differences in their climatic responses. For each PCA, components were retained until a cumulative variance of approximately 80% was explained. This threshold was met for *D. caudatum* (82.87%) and *P. incompleta* (82.58%). In the case of *C. macrocarpa*, two components explained 77.43% of the variance. While slightly below the threshold, this level was considered acceptable and consistent with the other two species, ensuring methodological uniformity across analyses. Variable importance within the PCA was assessed using the absolute value of component loadings. An inclusion threshold of |0.5| was applied to identify variables with substantial contributions to the retained components. A sensitivity check confirmed that no near-threshold values (e.g., 0.48–0.49) were present, reducing the risk of excluding climatically relevant variables. This approach allowed for a robust selection of variables while mitigating multicollinearity in subsequent modeling.

#### 4.3.4. Deterministic Population Projection Based on PCA-Derived Predictive Algorithm

To explore how climate variability influences population dynamics, we developed a heuristic demographic model informed by the results of PCA. The PCA was conducted to identify orthogonal axes of covariation among standardized climatic variables. Rather than using principal components directly as predictors in regression models, we used the component loadings to guide the construction of empirical climate indices. Specifically, we examined the loadings (eigenvectors) to identify climate variables that contributed strongly to the first two principal components and were ecologically meaningful based on theoretical reasoning or prior knowledge of species-specific sensitivities. These variables were then combined into composite expressions capturing additive and interactive effects of climatic stressors (e.g., temperature and moisture extremes) on population trajectories. For each species, an equation (structure in Equation (4)) was formulated using a small set of variables with PCA loadings higher or equal to |0.5|. The structure of each formula included multiplicative and additive terms to reflect hypothesized non-linear climatic interactions. Scalar coefficients were applied heuristically, not through formal regression or optimization, to ensure the resulting values aligned with the magnitude and variability observed in the empirical abundance data. These coefficients were adjusted to retain both interpretability and internal consistency across species.

(4) $$N_{t}=N_{t-1}-\left\{\left[{VE}_{PC1}\cdot \sum_{j}^{J}\alpha_{j}\cdot X_{j}\right]+\left[{VE}_{PC2}\cdot \sum_{j}^{J}\beta_{j}\cdot X_{j}\right]\right\}$$

where VE_PC1_ and VE_PC2_ represent the percentage of variance explained by principal components 1 and 2, respectively, and the coefficients α_j_ and β_j_ correspond to the loadings of variables X_j_ with absolute values greater than 0.5 in each respective component. The sums include only those variables whose loadings exceed this threshold, capturing the main climatic drivers identified by the PCA. This formulation has been compacted by extracting common minimum terms to streamline the model’s application and interpretation

The resulting equations function as conceptual climate impact indices: they are grounded in the multivariate structure revealed by PCA, but are not statistically fitted within a formal predictive modeling framework. These indices were incorporated into iterative, year-by-year population projection models to simulate temporal changes in abundance. This approach is explicitly exploratory and heuristic. While it does not include formal parameter estimation, uncertainty quantification, or account for other ecological processes (e.g., dispersal, biotic interactions), it offers a biologically grounded and interpretable method for hypothesis generation and for exploring directional responses to climatic variation. It also provides a structured foundation for future refinement as more comprehensive datasets become available through full dataset of the Andalusian Fern Recovery Plan [71].

#### 4.3.5. Deterministic Population Projection Based on Empirical Annual Change Rates

To evaluate the future demographic trajectory of the studied relict fern populations under stable environmental conditions, projection model grounded in observed interannual variation in total population size for each species was implemented. Specifically, we used annual census data collected between 2015 and 2022 to calculate empirical rates of population change. For this calculation, we excluded years in which reintroductions of individuals were documented, based on management records. This adjustment applies only to *P. incompleta*, for which introductions occurred in OGUs 8829 and 88,137 in 2015 and 2018, respectively. For *C. macrocarpa*, a single introduction was performed in OGU 8814 in 2014 (prior to the time series analyzed). No reintroductions were conducted in the *D. caudatum* populations included in this study during the analyzed period. These exclusions ensure that the calculated population change rates reflect intrinsic demographic dynamics rather than increases in individual numbers driven by conservation interventions. This approach assumes that differences in abundance between years reflect net mortality, and that survival can be approximated by the observed ratio of counts. For each year t, the annual rate of change r_t_ was computed using Equation (5):

(5) $$r_{t}=\frac{N_{t}-N_{t-1}}{N_{t-1}}$$

where N_t_ and N_t−1_ represent the number of individuals at years t and t−1, respectively. The arithmetic mean of the annual rates across the full census period was then calculated for each species (Equation (6)):

(6) $$R=\frac{1}{n-1}\sum_{t=2}^{n}r_{t}$$

This average change in abundance was used to project future population size in a recursive manner (Equation (7)):

(7) $$N_{t+1}=N_{t}\cdot (1-R)$$

This approach assumes that net annual change in abundance reflects a consistent underlying demographic process, and that such a process can be extrapolated under the assumption of stationary conditions. The projections were carried forward to 2050, assuming that key ecological drivers will remain like those observed during the baseline period. This modeling framework was applied independently for each species.

To incorporate uncertainty, stochastic projections were generated by sampling growth rates from the observed distribution. Empirical population trajectories were modeled deterministically using annual rates of population change observed during the baseline period (2014–2022), explicitly excluding years in which reinforcement activities were conducted. This approach aimed to simulate population dynamics under a no-intervention scenario, thereby providing a counterfactual baseline for evaluating the intrinsic demographic behavior and vulnerability of each species. Model performance was evaluated by comparing projected versus observed population sizes between 2015 and 2022. Four standard metrics were used to assess predictive accuracy: Mean Absolute Error (MAE), Root Mean Squared Error (RMSE), Mean Bias Error (MBE), and Mean Absolute Percentage Error (MAPE). To ensure robust estimation of accuracy and its variability, 100,000 bootstrap simulations for each time series were performed for each species, with summary statistics reported as mean ± standard deviation and 95% confidence intervals. All analyses were carried out using R version 4.4.2.

#### 4.3.6. Comparison and Validation of Projection Methods

To evaluate the validity and potential explanatory power of the projection models, we compared observed population abundances with model-predicted values for each species across the period 2016–2022. This comparison was conducted annually and independently for each modeling approach and fern species. For each year and species, the absolute difference between observed (field-sampled) and expected (model-predicted) abundance was calculated. This metric allowed us to quantify the residuals between observed and modeled dynamics. These residuals were not used for formal calibration but served as an exploratory assessment of model alignment with empirical patterns. These comparisons were used for two main purposes:

1. Retrospective model validation: By comparing modeled versus observed data during the last decade (2016–2022), we assessed the degree to which each model could reproduce historical population trajectories. Positive deviations (observed > expected) may suggest additional buffering factors (e.g., microhabitat protection, successful management), while negative deviations (observed < expected) might indicate unmodeled stressors or limited conservation effectiveness.
2. Model performance by species: This analysis allowed identification of which species were better captured by the modeling framework.

Additionally, for long-term forecasting, survival trajectories until 2050 were compared between the deterministic survival projection model based on the average annual rate of change and the heuristic PCA-informed projection model integrating climate interactions. This comparison aimed to highlight the degree of concordance or divergence between demographically and climatically driven projections under simplified assumptions. Both models are conceptual and not statistically fitted; their agreement or disagreement helps elucidate the potential role of recent demographic trends versus projected climatic pressures in shaping future population dynamics.

## 5. Conclusions

Populations of *C. macrocarpa*, *D. caudatum*, and *P. incompleta* in the mountain ranges near the Strait of Gibraltar, an area that serves as a genuine refuge for these paleomediterranean relict species, exhibit heterogeneous responses to climatological variables. This variability has allowed their classification into Operational Territorial Units that can serve as specific management units for conservation planning. Importantly, this study highlights that the impacts of climate change on these species are not uniform even across geographically close populations.

At the regional scale, considering all individuals across the studied populations, the projections developed indicate a decline in population sizes. When the effects of climate and projected climate change are included, an even more pronounced decrease emerges compared to that expected from demographic trends alone. Conservation measures must urgently continue and intensify, particularly introduction and reinforcement actions, to ensure the long-term viability of these species.

The observed differences in climate sensitivity among populations of the same species also underscore the need for future monitoring efforts at the microhabitat scale. Conditions such as temperature, humidity, and shade within the *canutos* may diverge substantially from those captured by coarse-resolution climate models or meteorological stations located in microenvironments that differ markedly from the studied sites. Integrating meteorological sensors using Internet of Things technology within fern populations, combined with continuous monitoring of herbivory pressure, could lay the groundwork for a short-term forecasting system to improve early detection of population declines and inform adaptive management. Over time, as data series grow and gaps in current records are filled, model estimations will become more robust, enhancing the effectiveness of conservation strategies.

Future studies should therefore prioritize high-resolution microclimatic monitoring and the development of predictive models that account for local variability and biotic interactions. Such approaches will help refine management actions and ensure these threatened, highly localized fern populations can persist under changing climatic conditions.

## Figures and Tables

**Table 1:** Pairwise Pearson correlation coefficients between annual abundance data for each population (Operational Geographical Unit, OGU) and standardized climatic variables over the 2014–2023 period. Significant correlations (*p* < 0.05) are highlighted in bold.

Population	Tmax	P	DmaxHW	DmaxCHD	ET
*Culcita macrocarpa*					
OGU01	0.44	0.13	−0.05	0.13	−0.39
OGU02	−0.08	0.30	**−0.69**	0.40	−0.62
OGU05	−0.16	−0.10	0.26	−0.34	−0.51
OGU06	−0.08	0.34	−0.22	0.39	**−0.66**
OGU08	−0.32	0.26	0.45	−0.03	**−0.67**
OGU09	−0.08	0.25	0.16	−0.04	−0.32
OGU10	0.20	−0.03	−0.45	0.09	**−0.66**
OGU11	0.21	0.09	−0.31	0.33	−0.05
OGU13	0.39	0.23	−0.11	0.28	−0.08
OGU15	0.39	0.05	0.19	−0.17	**−0.70**
*Pteris incompleta*					
OGU21	0.33	0.51	0.46	0.61	0.42
OGU22	**0.84**	0.38	**0.79**	0.56	0.41
OGU23	0.14	**0.86**	**0.98**	**0.68**	0.22
OGU24	0.14	0.69	**0.92**	0.61	0.05
OGU25	0.06	0.58	**0.88**	0.52	0.03
OGU27	**0.94**	**0.85**	0.20	0.35	0.39
OGU28	0.19	0.69	0.37	0.61	0.55
OGU29	0.29	0.80	**0.90**	**0.71**	0.02
OGU30	**0.87**	**0.86**	0.19	0.35	0.46
*Diplazium caudatum*					
OGU31	−0.27	0.09	−0.35	0.34	−0.42
OGU33	0.53	−0.39	−0.16	−0.22	**0.71**
OGU34	**−0.69**	0.47	0.19	0.28	−0.51
OGU35	0.42	−0.02	−0.46	0.24	0.33
OGU36	−0.24	0.26	0.25	−0.03	**−0.88**
OGU37	0.21	0.37	0.02	0.08	**−0.84**
OGU40	−0.22	0.05	0.09	0.03	**−0.72**
OGU41	−0.19	0.37	−0.06	0.34	**−0.72**
OGU44	−0.33	0.38	−0.05	0.34	**−0.81**
OGU45	−0.05	0.49	−0.14	0.39	−0.07

Climate variables: Tmax = Yearly maximum monthly mean air temperature measured at 2 m above the ground (°C); P = Annual cumulative precipitation (mm); DmaxHW = Maximum duration of heat waves per year, defined as at least 5 consecutive days exceeding the 90th percentile of a reference period (days); DmaxCHD = Maximum number of consecutive humid days per year, with daily precipitation above 1 mm (days); ET = Potential evapotranspiration (mm/month).

**Table 2:** Clustering of Operational Geographical Units (OGUs) into Operational Territorial Units (OTUs) based on similarity in their climate–abundance correlation profiles, using agglomerative hierarchical clustering (Ward’s method). The table also identifies the main climatic variables driving the grouping patterns.

spp.	Operational Territorial Unit	Operational Geographical Unit	Toponym	Variables Explaining Similarities in Climate–Abundance Relationships
*Culcita macrocarpa*	OTU01	OGU05	Garganta de la Vegueta	DmaxHW
		OGU08	Canuto de la Leña	
		OGU09	Canuto 6.7 Carril arriba	
		OGU15	Laja del Pinalejo	
	OTU02	OGU01	Linde Comares-Las Corzas	P, Tmax, DmaxCHD
		OGU11	Arroyo y Albinas del Viguetón	
		OGU13	Garganta del Niño	
	OTU03	OGU02	Canuto 6.4 Arriba Carril	ET, DmaxHW
		OGU06	Canuto 6.7 Carril abajo	
		OGU10	Juan de Sevilla	
*Pteris incompleta*	OTU04	OGU23	Albinas del Pino	ET, Tmax
		OGU24	Arroyo de Pepe Ayala 3	
		OGU25	Arroyo y Albinas del Pinillo	
		OGU29	Chorreras del Alto Mariscal	
	OTU05	OGU26	Arroyo del Pino	P, Tmax, DmaxCHD, DmaxHW
	OTU06	OGU27	Arroyo de Huerto Campano	DmaxHW
		OGU30	Garganta del Rayo	
	OTU07	OGU21	Junta afluentes 6.1-6.4	DmaxCHD
		OGU22	Arroyo Pepe Ayala 1	
		OGU28	Albina y Alto Mariscal	
*Diplazium caudatum*	OTU08	OGU33	Comares	ET, Tmax
		OGU35	Pedregoso	
	OTU09	OGU31	Pedregoso	DmaxCHD
		OGU45	Ojén	
	OTU10	OGU34	Pedregoso	ET
		OGU36	Pedregoso	
		OGU37	Ojén	
		OGU40	Pedregoso	
		OGU41	Pedregoso	
		OGU44	Pedregoso	

Climate variable abbreviations: Tmax = Yearly maximum monthly mean air temperature (°C); P = Annual cumulative precipitation (mm); DmaxHW = Maximum duration of heat waves (days); DmaxCHD = Maximum number of consecutive humid days (days); ET = Potential evapotranspiration (mm/month).

**Table 3:** Loadings of climatic variables on the first two principal components (PC1 and PC2) and percentage of variance explained for each species in the PCA of climate–abundance correlations.

Species	Principal Component	% Variance Explained	Variable	Loading PC1	Loading PC2
*Culcita macrocarpa*	1	52.32%	Tmax	0.25	0.73
			P	0.10	−0.20
			DmaxHW	−0.79	0.24
			DmaxCHD	0.52	−0.14
			ET	0.18	0.58
	2	25.11%	Tmax	0.25	0.73
			P	0.10	−0.20
			DmaxHW	−0.79	0.24
			DmaxCHD	0.52	−0.14
			ET	0.18	0.58
*Pteris incompleta*	1	54.19%	Tmax	0.76	0.15
			P	0.13	0.18
			DmaxHW	−0.39	0.75
			DmaxCHD	−0.04	0.50
			ET	0.51	0.36
	2	28.68%	Tmax	0.76	0.15
			P	0.13	0.18
			DmaxHW	−0.39	0.75
			DmaxCHD	−0.04	0.50
			ET	0.51	0.36
*Diplazium caudatum*	1	57.41%	Tmax	−0.46	0.06
			P	0.53	0.19
			DmaxHW	0.37	−0.66
			DmaxCHD	0.35	0.71
			ET	−0.50	0.15
	2	25.17%	Tmax	−0.46	0.06
			P	0.53	0.19
			DmaxHW	0.37	−0.66
			DmaxCHD	0.35	0.71
			ET	−0.50	0.15

**Table 4:** Comparison between the number of individuals observed and modeled based on climatic conditions from 2016 to 2022. Positive differences (Observed > Modeled) indicate effective conservation, while negative differences indicate impacts not buffered by conservation measures.

	2016	2017	2018	2019	2020	2021	2022
*Culcita macrocarpa*							
Observed	380	359	375	373	377	363	360
Modeled	370	349	345	370	373	370	352
Difference (O–M)	10	10	30	3	4	−7	8
*Pteris incompleta*							
Observed	312	456	535	613	582	547	494
Modeled	300	302	471	549	612	583	549
Difference (O–M)	12	154	64	64	−30	−36	−55
*Diplazium caudatum*							
Observed	233	397	405	438	421	379	366
Modeled	224	221	391	398	440	417	357
Difference (O–M)	9	176	14	40	−19	−38	9

**Table 5:** Summary of model performance metrics comparing observed and modeled individual counts for three species over 2016–2022. Metrics include Mean Absolute Error (MAE), Root Mean Squared Error (RMSE), Mean Bias Error (MBE) and Mean Absolute Percentage Error (MAPE).

Species	MAE	RMSE	MBE	MAPE
*Culcita macrocarpa*	10.29	13.54	8.29	2.78
*Pteris incompleta*	59.29	73.50	24.71	11.8
*Diplazium caudatum*	43.57	70.4	27.32	11.1

**Table 6:** Summary of model performance metrics comparing observed and projected annual population counts for three fern species between 2016 and 2022. Metrics were computed annually across 100,000 bootstrap simulations derived from empirical survival rates. Values represent the mean ± standard deviation, and the 2.5% and 97.5% quantiles of the empirical distribution, thus reflecting 95% confidence intervals. Performance was assessed using Mean Absolute Error (MAE), Root Mean Squared Error (RMSE), Mean Bias Error (MBE), and Mean Absolute Percentage Error (MAPE).

Species	MAE	RMSE	MBE	MAPE (%)	
*Culcita macrocarpa*	46.63 ± 26.67 [11.24–116.34]	56.7 ± 32.69 [15.79–130.46]	40.14 ± 35.92 [19.25–116.28]	12.95 ± 8.18 [3.26–32.22]	
*Pteris incompleta*	188.68 ± 12.60 [162.64–211.64]	212.82 ± 13.64 [184.42–237.58]	188.63 ± 12.65 [162.45– 211.64]	47.92 ± 3.31 [41.11–53.97]	
*Diplazium caudatum*	282.25 ± 30.18 [210.52–323.44]	326.79 ± 32.48 [248.71–371.38]	281.38 ± 31.08 [207.54–323.44]	52.84 ± 5.88 [38.95–60.82]	

**Table 7:** General description of the study species: *C. macrocarpa*, *Diplazium caudatum*, and *Pteris incompleta*. The table presents the family to which each species belongs, their distribution areas, the ratio of species within the same family at the global level versus the Mediterranean region and Europe, chromosome number, spore type (m = monolete; t = trilete), and conservation status. D23 refers to Decree 23/2012, LRFA to the Red List of the Vascular Flora of Andalusia 2005, and LRFE to the Red List of the Spanish Vascular Flora 2008. Conservation categories include CR (Critically Endangered), EN (Endangered), and VU (Vulnerable).

	Family	General Distribution	Ratio nº spp.	Nº crom.	Spores (m, t)	Category of Threat
*Diplazium caudatum* (Cav.) Jermy	Athyriaceae (Polypodiales)	Aljibic Sector and Macaronesian Region	2/350	82	m	D23: EN. LRFA: CR. LRFE: CR.
*Culcita macrocarpa* C.Presl	Culcitaceae (Cyatheales)	Aljiblical Sector, Cantabro-Atlantic Subprovince and Macaronesian Region.	1/002	136	t	D23: EN. LRFA: CR. LRFE: EN.
*Pteris incompleta* Cav.	Pteridaceae (Polypodiales)	Aljibic sector, Tingitana Peninsula and Macaronesian Region.	3/250	58	t	D23: EN. LRFA: CR LRFE: VU.

**Table 8:** Description of the climatic variables considered.

Variable	Definition	Units	Ecological Roles in Ferns
Tmax	Yearly maximum monthly mean air temperature measured at 2 m above ground	°C	Paleomediterranean ferns require high and stable temperatures that resemble the subtropical climatic conditions that prevailed in their habitats in the past [15,69].
P	Annual cumulative precipitation	mm	Essential for completing the life cycle (sporophyte and gametophyte phases); all three species depend on high atmospheric and soil moisture [41].
DmaxHW	Maximum duration of heat waves per year (≥5 consecutive days above the 90th percentile)	days	Potentially critical for fern populations; ferns are especially vulnerable to prolonged heat and dryness [69], but microclimatic conditions in canutos may buffer this effect [15].
DmaxCHD	Maximum number of consecutive humid days per year (days with daily precipitation >1 mm)	days	Essential for reproductive processes. Consecutive humid periods facilitate spore germination and gametophyte development [15,69,70]
ET	Potential evapotranspiration, estimated by the Thornthwaite method (k = 0.69)	mm/month	Key indicator of drought stress; high values reflect greater atmospheric demand for water, leading to negative effects on ferns due to their strong dependence on moisture [41].

## Data Availability

Data will be available upon request, subject to prior consultation with the Consejería de Sostenibilidad, Medio Ambiente y Economía Azul, Junta de Andalucía, due to data confidentiality.

## Supplementary Materials

The following supporting information can be downloaded at: https://www.mdpi.com/article/10.3390/plants14152380/s1. References [76,77,78] are cited in the supplementary materials.
