# Supplementary material for: Projecting Extinction Risk and Assessing Conservation Effectiveness for Three Threatened Relict Ferns in the Western Mediterranean Basin
Source: Plants (Basel). 2025 Aug 1;14(15):2380. doi: 10.3390/plants14152380 (PMC12348807; doi:10.3390/plants14152380)
Supplement: Supplementary file 1 [file plants-14-02380-s001.zip › plants-3736830-supplementary.pdf]

Projecting Extinction Risk and Assessing Conservation Effectiveness for Three Threatened Relict Ferns in the Western Mediterranean Basin

Supplementary Material

Equation S1. The statistical model employed follows the Latent Gaussian Model (LGM) framework, which is a specific case of Bayesian Hierarchical Models (BHMs) characterized by an additive structure in the linear predictor and an observation process that depends only on this predictor and the parameters of the selected likelihood. The model was implemented in R-INLA v.24.12.11 [1]. This model captures both the spatial variability among populations and the heterogeneity in covariate effects on them, as well as temporal correlation (AR(1)) in the abundance data. The inclusion of these random effects is necessary to avoid biased estimates of covariate effects arising from the hierarchical structure of the data and residual temporal autocorrelation [2]. For the fixed effects in the model, Gaussian prior distributions with a mean of zero and a standard deviation of 1000 were used. These priors represent weakly informative specifications that allow the data to dominate the posterior distributions. Penalized Complexity (PC) priors were implemented for precision parameters following the framework developed by [3]. PC priors are designed to penalize model complexity by favoring simpler model structures over more complex ones. For the random intercepts associated with each Operational Geographical Unit (OGU), the PC prior was specified so that there is a 0.1 probability that the standard deviation exceeds 1. For the random slopes of each variable, a PC prior was set so that there is a 0.1 probability that the standard deviation exceeds 0.5. For the autoregressive process, the PC prior was defined so that there is a 0.9 probability that the standard deviation is less than 0.1. The autoregressive order 1 (AR(1)) structure associated with temporal effects was assigned a Penalized Complexity prior. This prior specification assumes a 0.9 probability that the temporal autocorrelation coefficient is greater than zero

Let $\boldsymbol{Y_{ij}}$ be the abundance observed in OGU i in time t (with i = 1, …, I; t = 1, …, T).

$$Y_{it} \sim Poisson(\lambda_{it})$$

$$log(\lambda_{it}) = \eta_{it}$$

where the linear predictor η_{it} is specified as:

$$\eta_{it} = \beta_0 + \beta_{0[i]} + \sum_{k=1}^{5} \beta_k \cdot X_{k,it} + \sum_{k=1}^{5} \beta_{k[i]} \cdot X_{k,it} + u_{t,i}$$

Random effects:

1. Random intercept by OGU: $\beta_{0[i]} \sim Normal(0, \sigma)$, $\sigma \sim PC(1, 0.1)$
2. Random slopes by OGU: $\beta_{k[i]} \sim Normal(0, \sigma_k)$, $\sigma \sim PC(0.5, 0.1)$
3. Replicated autoregressive process of order 1 (AR(1)), with replication by OGU and shared hyperparameters: $u_{t,i} = \rho \cdot u_{t-1,i} + \varepsilon_t$, $\varepsilon_t \sim Normal(0, 1)$, $\rho \sim PC(0.1, 0.9)$

Table S1. Estimates of linear fixed effects and model hyperparameters. Results are presented as the mean and the 95% credible interval, defined by the 0.025 and 0.975 quantiles of the respective posterior distributions. Fixed-effect estimates are reported on the linear predictor scale (log-scale), with covariates standardized (z-scored) to allow for comparison of effect sizes.

Tmax = Yearly maximum monthly mean air temperature measured at 2 meters above the ground (°C); P = Annual cumulative precipitation (mm); DmaxHW = Maximum duration of heat waves per year, defined as at least 5 consecutive days exceeding the 90th percentile of a reference period (days); DmaxCHD = Maximum number of consecutive humid days per year, with daily precipitation above 1 mm (days); ET = Potential evapotranspiration (mm/month)

		Estimate
Fixed effect		
	Intercept	3.03 [2.577, 3.47]
	Tmax	0.02 [-0.076, 0.106]
	P	-0.01 [-0.078, 0.065]
	DmaxCHD	0.04 [-0.003, 0.083]
	DmaxHV	0.07 [0.005, 0.138]
	ET	-0.12 [-0.217, -0.032]
Hyperparmater		
	Standard deviation of random intercepts by OGU	1.23 [0.972, 1.546]
	Standard deviation of random slopes for Tmax by OGU	0.21 [0.147, 0.301]
	Standard deviation of random slopes for P by OGU	0.13 [0.087, 0.185]
	Standard deviation of random slopes for DmaxCHD by OGU	0.01 [0.003, 0.091]
	Standard deviation of random slopes for DmaxHW by OGU	0.04 [0.01, 0.104]
	Standard deviation of random slopes for ET by OGU	0.22 [0.145, 0.316]
	Standard deviation of the AR(1) process	1.27 [0.987, 1.64]
	Temporal autocorrelation of the AR(1) process	0.988 [0.979, 0.994]

Figure S1. Total effect of yearly maximum monthly mean air temperature for each Operational Geographical Unit (OGU). The effect is calculated as the sum of the posterior distributions of the fixed effect of the covariate and the random effect for each OGU. Results are presented using similar colors but with different shades and palettes according to the membership of each OGU within its respective Operational Territorial Unit (OTU) and species. The figure shows the mean and the 50%, 75%, and 95% credible intervals of the posterior distributions of the total effect. Where the vertical dashed line, representing no temperature effect on abundance, is outside a given interval, it can be interpreted that there is evidence for an effect of the magnitude indicated on the X-axis with that level of probability. Thus, for a given estimate, there may be evidence of an effect at lower probability intervals (e.g., 50% or 75%) but not at 95%, the latter providing the highest confidence in the model estimates. These results reinforce and are consistent with those presented in section *"2.1. Correlation between climate and demographic data"*, highlighting the variability in responses to climatic variables among OGUs and, to a lesser extent, among those grouped within the same OTUs.

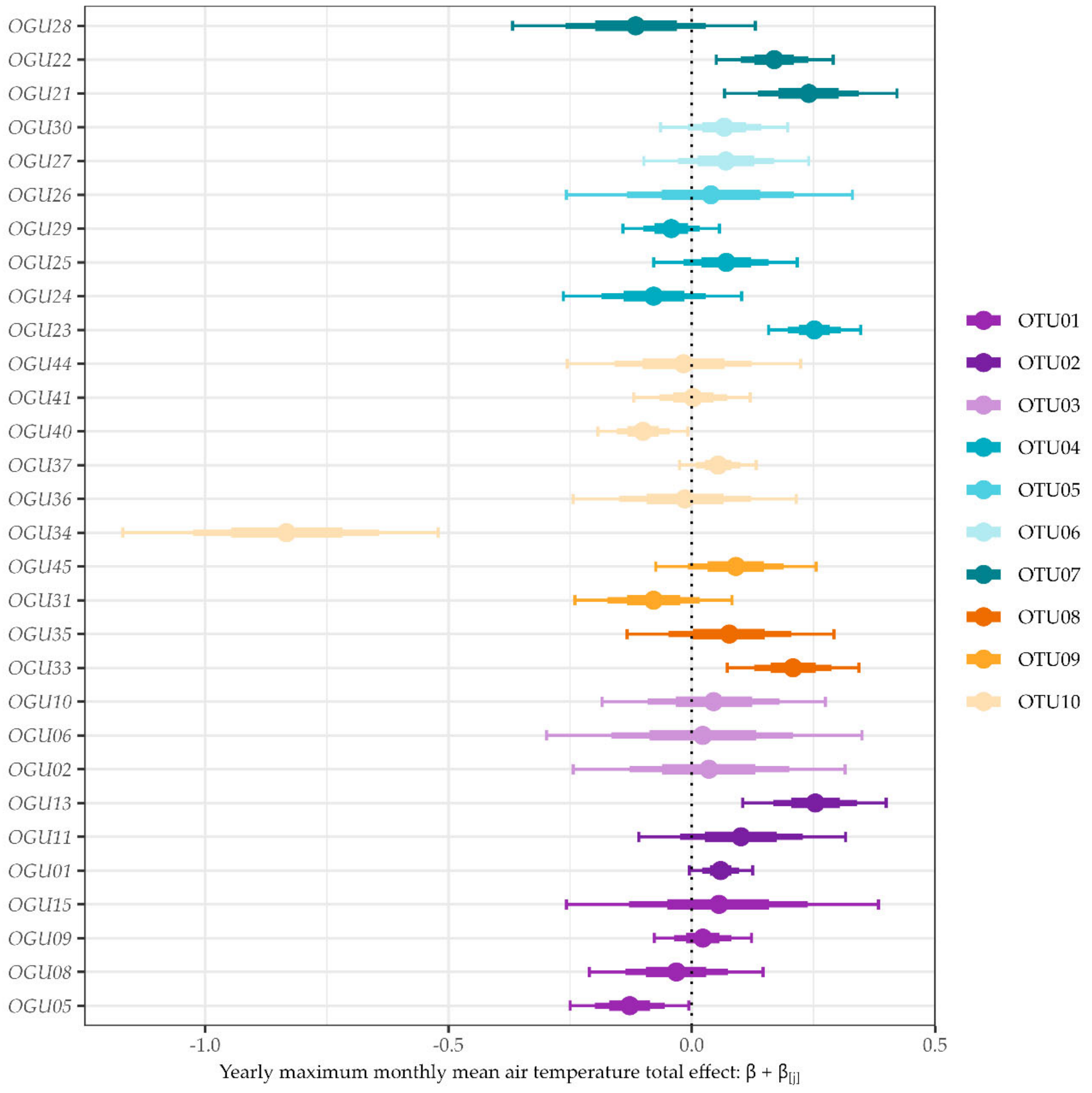

Figure S2. Total effect of annual cumulative precipitation for each Operational Geographical Unit (OGU). The effect is calculated as the sum of the posterior distributions of the fixed effect of the covariate and the random effect for each OGU. Results are presented using similar colors but with different shades and palettes according to the membership of each OGU within its respective Operational Territorial Unit (OTU) and species. The figure shows the mean and the 50%, 75%, and 95% credible intervals of the posterior distributions of the total effect. Where the vertical dashed line, representing no temperature effect on abundance, is outside a given interval, it can be interpreted that there is evidence for an effect of the magnitude indicated on the X-axis with that level of probability. Thus, for a given estimate, there may be evidence of an effect at lower probability intervals (e.g., 50% or 75%) but not at 95%, the latter providing the highest confidence in the model estimates. These results reinforce and are consistent with those presented in section *"2.1. Correlation between climate and demographic data"*, highlighting the variability in responses to climatic variables among OGUs and, to a lesser extent, among those grouped within the same OTUs.

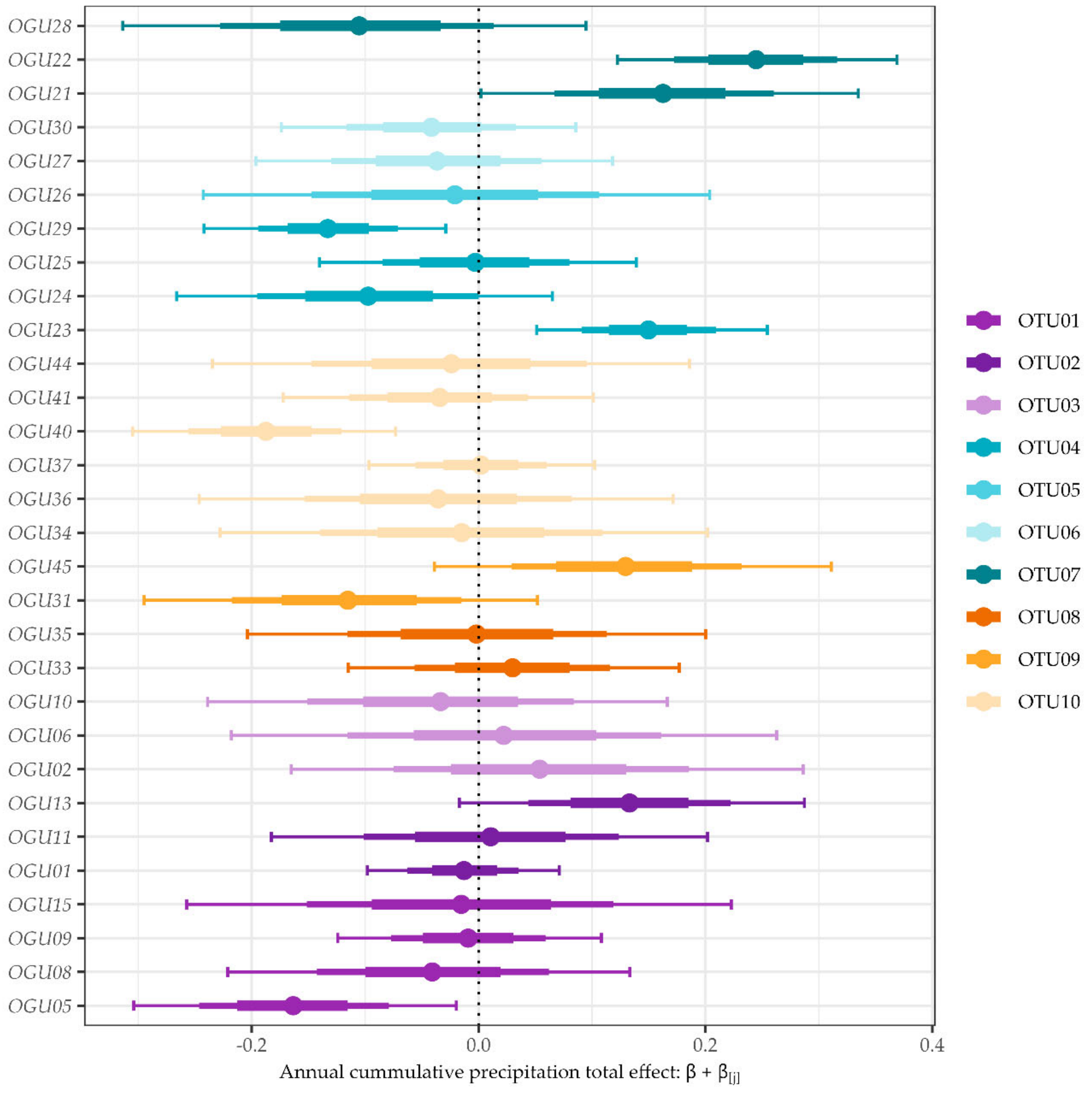

Figure S3. Total effect of maximum duration of heatwaves per year for each Operational Geographical Unit (OGU). The effect is calculated as the sum of the posterior distributions of the fixed effect of the covariate and the random effect for each OGU. Results are presented using similar colors but with different shades and palettes according to the membership of each OGU within its respective Operational Territorial Unit (OTU) and species. The figure shows the mean and the 50%, 75%, and 95% credible intervals of the posterior distributions of the total effect. Where the vertical dashed line, representing no temperature effect on abundance, is outside a given interval, it can be interpreted that there is evidence for an effect of the magnitude indicated on the X-axis with that level of probability. Thus, for a given estimate, there may be evidence of an effect at lower probability intervals (e.g., 50% or 75%) but not at 95%, the latter providing the highest confidence in the model estimates. These results reinforce and are consistent with those presented in Section 2.1, *Correlation between climate and demographic data*. While some negative correlations were observed when calculating linear correlations independently for each variable, the vast majority were positive. The model-based estimates presented in this figure indicate an effect that, although associated with high uncertainty, is consistently positive across OGUs. Therefore, these results do not contradict those reported in the main manuscript but rather complement and support them.

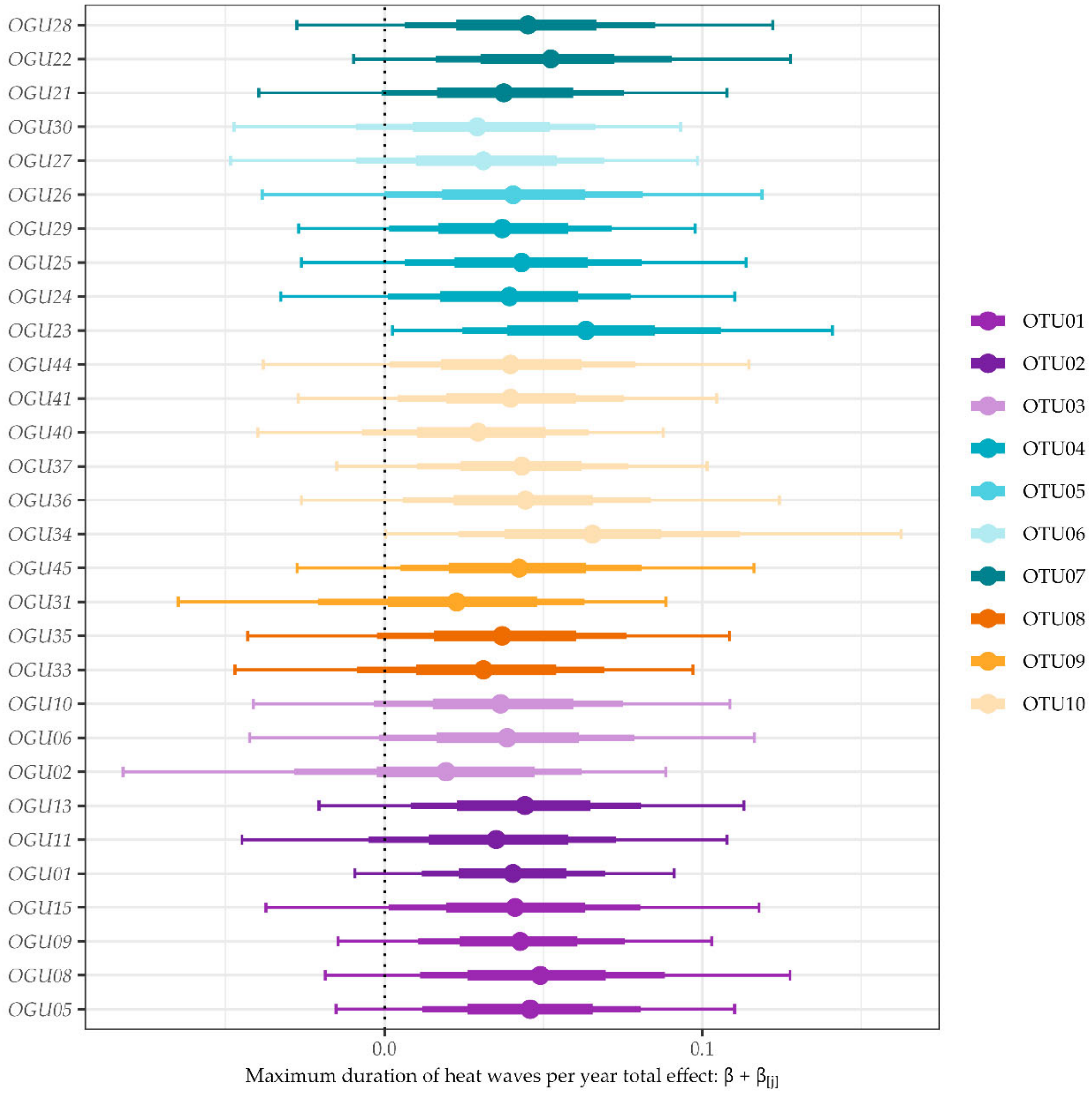

Figure S4. Total effect of maximum number of consecutive humid days for each Operational Geographical Unit (OGU). The effect is calculated as the sum of the posterior distributions of the fixed effect of the covariate and the random effect for each OGU. Results are presented using similar colors but with different shades and palettes according to the membership of each OGU within its respective Operational Territorial Unit (OTU) and species. The figure shows the mean and the 50%, 75%, and 95% credible intervals of the posterior distributions of the total effect. Where the vertical dashed line, representing no temperature effect on abundance, is outside a given interval, it can be interpreted that there is evidence for an effect of the magnitude indicated on the X-axis with that level of probability. Thus, for a given estimate, there may be evidence of an effect at lower probability intervals (e.g., 50% or 75%) but not at 95%, the latter providing the highest confidence in the model estimates. These results reinforce and are consistent with those presented in Section 2.1, *Correlation between climate and demographic data*. While some negative correlations were observed when calculating linear correlations independently for each variable, the vast majority were positive. The model-based estimates presented in this figure indicate an effect that, although associated with high uncertainty, is consistently positive across OGUs. Therefore, these results do not contradict those reported in the main manuscript but rather complement and support them.

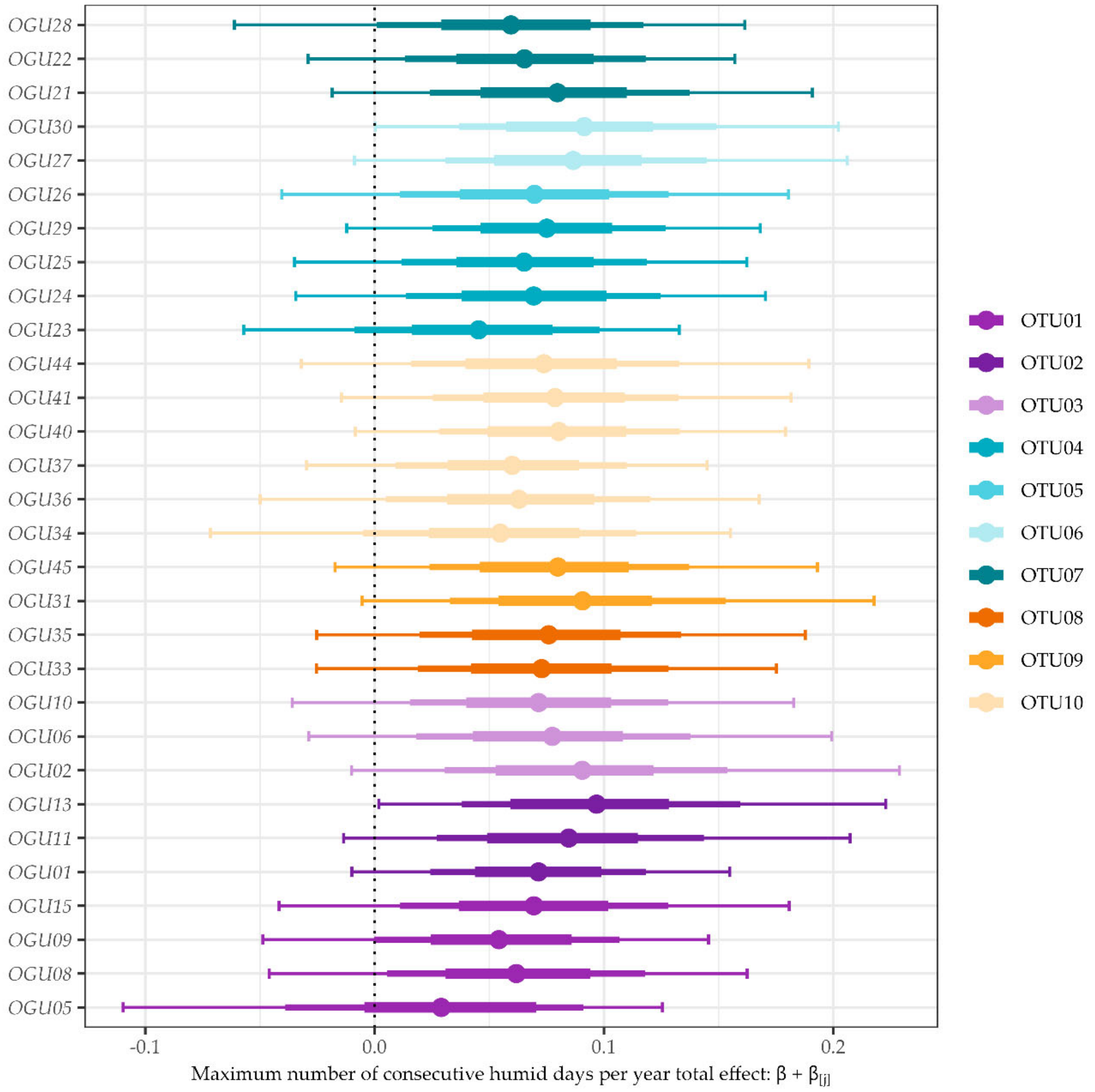

Figure S5. Total effect of potential evapotranspiration for each Operational Geographical Unit (OGU). The effect is calculated as the sum of the posterior distributions of the fixed effect of the covariate and the random effect for each OGU. Results are presented using similar colors but with different shades and palettes according to the membership of each OGU within its respective Operational Territorial Unit (OTU) and species. The figure shows the mean and the 50%, 75%, and 95% credible intervals of the posterior distributions of the total effect. Where the vertical dashed line, representing no temperature effect on abundance, is outside a given interval, it can be interpreted that there is evidence for an effect of the magnitude indicated on the X-axis with that level of probability. Thus, for a given estimate, there may be evidence of an effect at lower probability intervals (e.g., 50% or 75%) but not at 95%, the latter providing the highest confidence in the model estimates. These results reinforce and are consistent with those presented in section *"2.1. Correlation between climate and demographic data"*, highlighting the variability in responses to climatic variables among OGUs and, to a lesser extent, among those grouped within the same OTUs.

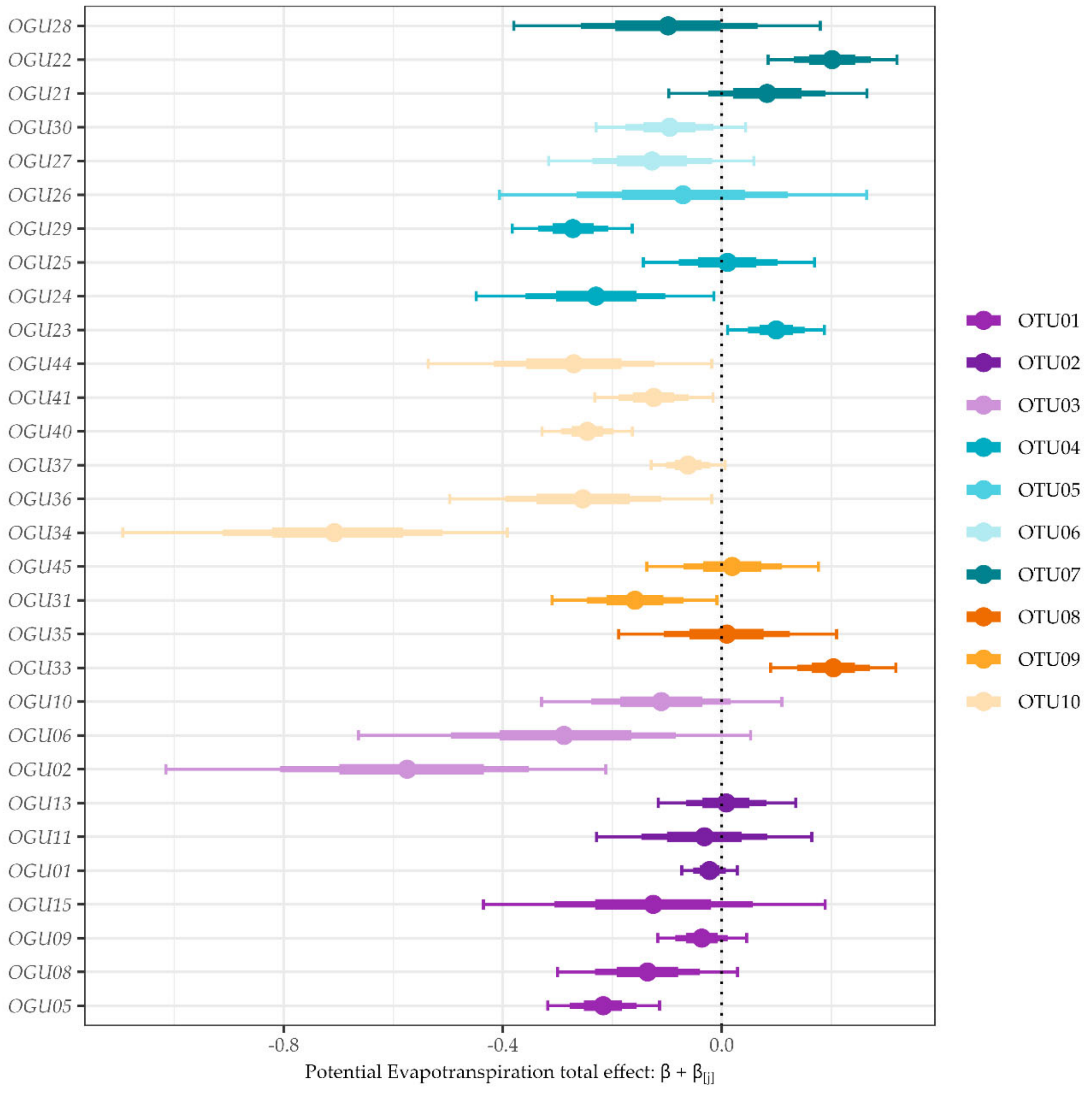

References

1. Rue, H.; Martino, S.; Chopin, N. Approximate Bayesian Inference for Latent Gaussian Models by Using Integrated Nested Laplace Approximations. *Journal of the Royal Statistical Society Series B: Statistical Methodology* **2009**, *71*, 319–392, doi:10.1111/j.1467-9868.2008.00700.x.

2. Banerjee, S.; Carlin, B.P.; Gelfand, A.E. Hierarchical Modeling and Analysis for Spatial Data; 2nd ed.; *Chapman and Hall/CRC: New York*, **2014**; ISBN 978-0-429-13717-4.

3. Simpson, D.; Rue, H.; Riebler, A.; Martins, T.G.; Sørbye, S.H. Penalising Model Component Complexity: A Principled, Practical Approach to Constructing Priors. *Statistical Science* **2017**, *32*, 1–28, doi:10.1214/16-STS576.
